# Whole Exome Sequencing-Based Identification of a Novel Gene Involved in Root Hair Development in Barley (*Hordeum vulgare* L.)

**DOI:** 10.3390/ijms222413411

**Published:** 2021-12-14

**Authors:** Katarzyna Gajek, Agnieszka Janiak, Urszula Korotko, Beata Chmielewska, Marek Marzec, Iwona Szarejko

**Affiliations:** 1 Institute of Biology, Biotechnology and Environmental Protection, Faculty of Natural Sciences, University of Silesia, 40-032 Katowice, Poland; katarzyna.gajek@us.edu.pl (K.G.); agnieszka.janiak@us.edu.pl (A.J.); beata.chmielewska@us.edu.pl (B.C.); marek.marzec@us.edu.pl (M.M.); 2 Centre for Bioinformatics and Data Analysis, Medical University of Bialystok, 15-089 Bialystok, Poland; urszula.korotko@umb.edu.pl

**Keywords:** *Hordeum vulgare*, barley, root hairs, tip growth, plant cell wall, xyloglucan, *HvCSLC1*, exome capture

## Abstract

Root hairs play a crucial role in anchoring plants in soil, interaction with microorganisms and nutrient uptake from the rhizosphere. In contrast to Arabidopsis, there is a limited knowledge of root hair morphogenesis in monocots, including barley (*Hordeum vulgare* L.). We have isolated barley mutant *rhp1.e* with an abnormal root hair phenotype after chemical mutagenesis of spring cultivar ‘Sebastian’. The development of root hairs was initiated in the mutant but inhibited at the very early stage of tip growth. The length of root hairs reached only 3% of the length of parent cultivar. Using a whole exome sequencing (WES) approach, we identified G1674A mutation in the *HORVU1Hr1G077230* gene, located on chromosome 1HL and encoding a cellulose synthase-like C1 protein (HvCSLC1) that might be involved in the xyloglucan (XyG) synthesis in root hairs. The identified mutation led to the retention of the second intron and premature termination of the HvCSLC1 protein. The mutation co-segregated with the abnormal root hair phenotype in the F_2_ progeny of *rhp1.e* mutant and its wild-type parent. Additionally, different substitutions in *HORVU1Hr1G077230* were found in four other allelic mutants with the same root hair phenotype. Here, we discuss the putative role of HvCSLC1 protein in root hair tube elongation in barley.

## 1. Introduction

Root hairs (RHs) are long tubular outgrowths of root epidermal (rhizodermal) cells that play a role in absorbing water and nutrients from the soil. They increase significantly root surface; for example, it was estimated in barley that the root surface can be expanded by 112–245%, depending on the root hair length of the cultivar [1]. Their role is especially important when plants grow under stressful conditions, such as phosphorus deficiency or drought [2,3]. Root hairs are also involved in interaction with the soil microorganisms [4]. It was proved that they play a crucial role during formation of nitrogen-fixing nodules by symbiotic *Rhizobiaceae* members in legumes [5,6].

Root hair morphogenesis includes three main stages: (1) rhizodermal cell fate specification, (2) root hair initiation and bulge formation, (3) elongation of a root hair tube. Each root hair is formed by one special epidermal cell called a trichoblast [7], while a cell without potential for hair forming is named an atrichoblast [8,9]. The similar proportion of trichoblasts and atrichoblasts within the rhizodermis was observed in many plant species, including cereals [10]. The molecular basis of root hair morphogenesis has been relatively well described in Arabidopsis, e.g., in a recent review on rhizodermal cell fate determination and hormonal/environmental control of root hair growth [11] and an extensive review on the tip growth of the root hairs [12]. However, there is a limited knowledge on molecular mechanisms controlling root hair morphogenesis in monocots, including barley.

Contrary to the different rhizodermis patterning in dicots and monocots, the further stages of root hair formation, including initiation and elongation of root hairs, are common for both plant clades [13]. Root hairs elongate by tip growth, characteristic also for elongation of pollen tubes and fungal phyphae [14]. The tip growth of root hairs is accomplished through the following steps: (1) loosening of the cell wall, (2) induction of the synthesis of cell wall polymers and formation of the cellulose–hemicellulose network, (3) reorganization of the cytoskeleton and vesicle transport [13,15]. These steps require the involvement of a complex network of genes that were best studied in Arabidopsis (for review, see [16]). Most of the identified genes encode proteins involved in cell wall modification during tip growth, but the precise role of the particular cell wall components in the elongation of root hair has not always been determined.

In monocots, only 14 genes related to root hair elongation were reported using mutants or overexpression lines, most of them in *Oryza sativa* [17]. Three rice genes encoding expansins, *OsEXPA8*, *OsEXPA17*, *OsEXPB2* were shown to be involved in the cell wall loosening, the initial step of root hair tube elongation [18,19,20]. Two further genes identified in rice: *OsCSLD1* (*CELLULOSE SYNTHASE-LIKE D1*) and *OsMOGS* (*MANNOSYL-OLIGOSACCHARIDE GLUCOSIDASE*) encode proteins that are crucial for the synthesis of cellulose in the elongating wall during tip growth [21,22]. Moreover, the *OsXXT1* gene encoding xyloglucan 6-xylosyltransferase responsible for xyloglucan (XyG) biosynthesis is also involved in forming cellulose/hemicellulose network at the growing tip [23]. Another gene that is important in the elongation of root hair tube in rice is *OsSNDP1* (*SEC14-NODULIN DOMAIN PROTEIN*) encoding a protein related to vesicle transport and organization of the cytoskeleton [24]. In turn, *OsFH1* (*FORMIN HOMOLOGY 1*) encoding a formin-like protein is involved in polymerization of the actin cytoskeleton [25]. It is also known that two genes, *OsRHL1* (*ROOT HAIRLESS 1*), encoding a bHLH transcription factor, and *OsRTH1*/*OsAPY1* (*REVERSION-TO-ETHYLENE SENSITIVITY 1 HOMOLOG 1*/*APYRASE 1*), encoding an apyrase protein, are necessary for root hair elongation [26,27]. However, the molecular function of these genes during root hair development remains to be resolved. Interestingly, mutants carrying nucleotide substitutions in the genes presented above (*OsEXPA17*, *OsEXPB2*, *OsCSLD1*, *OsMOGS OsXXT1*, *OsSNDP1*, *OsFH1*, *OsRHL1*, *OsRTH1*/*OsAPY1*) show highly reduced length of root hairs, whereas overexpression of *OsEXPA8* leads to the development of longer root hairs compared to the wild type.

Furthermore, in *Zea mays*, four genes that control root hair elongation were reported. The model of the functional link between *RTH5*, *RTH6* and *RTH3* (*ROOT HAIRLESS 5, 6, 3*) genes has been proposed, showing the molecular mechanism underlying root hair tube elongation and the involvement of these genes in the particular processes. Activity of *RTH5*, *RTH6* and *RTH3* allows to initiate and maintain tip growth by modification of the cell wall and synthesis of the cellulose microfibrils [28]. The *RTH5* gene encodes a NADPH oxidase involved in the loosening of the cell wall by producing reactive oxygen species (ROS) in the root hair tip [29]. In turn, the *RTH6* gene was shown to encode a monocot-specific cellulose synthase CSLD5 required for the production of cellulose during tube growth [28]. Finally, the *RTH3* gene is responsible for the organization of cellulose microfibrils [30]. In addition to these genes, the role of *RTH1* encoding a protein involved in polar exocytosis during root hair elongation has been reported [31]. These data suggest a key role of genes that control the cell wall formation in root hair development and growth of the root hair tube. Similar to rice, maize plants carrying mutations in identified genes displayed the altered root hair phenotype.

The primary cell wall of root hairs and other growing cells is both a rigid and flexible structure. It is characterized by tensile strength as well as being extensibility required for the volume increase. Primary plant walls are composed of a cellulose microfibril network embedded in a matrix of hemicelluloses, pectins and structural proteins [32]. The cellulose microfibrils are synthesized at the plasma membrane, but the matrix polysaccharides are made in the Golgi bodies and then assembled in the cell wall by exocytosis [33]. The primary cell wall of root hair is a thin layer at the tip that is developed by turgor pressure. To allow extension of this layer during root hair tube elongation, it is necessary to provide new cell wall ingredients such as cellulose microfibrils, hemicelluloses, pectins and proteins to the hair tip [28,34]. Literature data indicate that the organization of the glycans in the wall determines the direction of the cell expansion [35]. The primary cell wall at the tip of root hair is expanded when cellulose microfibrils are randomly arranged [36].

Mutagenized plant populations are a useful tool in functional genomics for identification of genes underlying phenotypes of interest [37]. Using TILLING (Targeted Induced Local Lesions In Genomes), a reverse genetics strategy to detect mutations in a target gene, further studies are required to link the causative mutation with the observed phenotypic change. These studies include crossing of the mutant and its wild type parent, followed by co-segregation analysis, which makes the whole procedure labor- and time-consuming. The forward genetics method that includes linkage mapping and map-based isolation of a gene of interest is even more labor-intensive. However, the advances of the next generation sequencing (NGS) technologies opened the way to accelerate both TILLING and map-based cloning strategies [38]. Screening of mutagenized individuals in conjunction with high-throughput sequencing can facilitate the identification of a gene responsible for the mutant phenotype. The exome sequencing approach known as a whole exome sequencing (WES), representing target capture and sequencing, has already been successfully applied, for identification of genetic variation in mutagenized individuals of rice and wheat [38,39,40].

Although several barley mutants with different changes of root hair phenotype have been reported [41], the sequence of one gene only has been isolated till now. We have identified the *HvRhl1* (*H. vulgare ROOT HAIRLESS 1*) gene encoding a bHLH (basic-helix-loop-helix) transcription factor with LRL (LOTUS JAPONICUS ROOTHAIRLESS1-LIKE) domain that plays a crucial role at the early stage of root hair morphogenesis, during rhizodermal cell differentiation [42]. Mutation in this gene causes a change of rhizodermis patterning that results in the completely root hairless phenotype. In present studies, we have isolated the *rhp1.e* (*root hair primordium 1.e*) mutant with extremely short root hairs in a barley TILLING population developed after chemical mutagenesis of the spring cultivar ‘Sebastian’. Using a whole exome sequencing workflow, we have identified a candidate gene—*HvCSLC1* (*H. vulgare CELLULOSE SYNTHASE-LIKE C1*) carrying a mutation responsible for this phenotype. We have confirmed the role of *HvCSLC1* in root hair development by additional allelic mutations and co-segregation of the mutations with the root hair phenotype. This gene encodes a glucan synthase that might be involved in xyloglucan (XyG) biosynthesis in barley. Our results reveal the role of *HvCSLC1* during root hair tube elongation, indicating the importance of the cellulose synthase-like C enzyme activity in walls of the growing root hairs of barley.

## 2. Results

### 2.1. Isolation and Phenotyping of the rhp1.e Mutant

**Figure 1:**
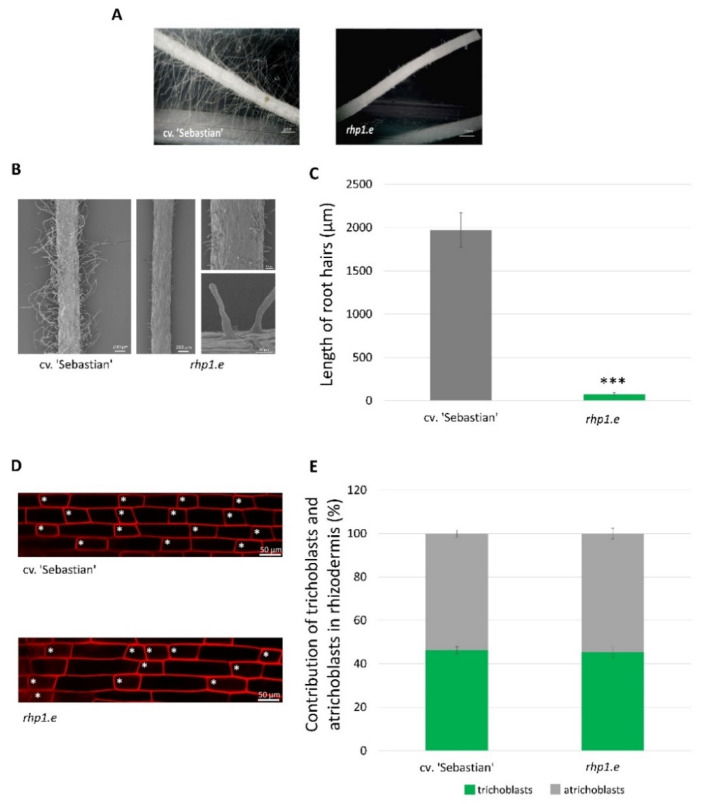
Root hair phenotype of WT cv. ‘Sebastian’ and *rhp1.e* mutant. (**A**) Light microscope and (**B**) SEM images of the root hair zone of WT and *rhp1.e.* (**C**) Length of the root hairs in the WT and mutant. Significant differences were calculated using Student’s *t*-test (*** *p* < 0.001). Graph shows mean of *n* = 500 root hairs per each genotype with SD. (**D**) Rhizodermis patterning in WT cv. ‘Sebastian’ and *rhp1.e* mutant. Optical sections through the rhizodermal layer and proportions of the two types of cell present in the root epidermis. Stars indicate trichoblasts. (**E**) Proportion of trichoblasts and atrichoblasts in rhizodermis of WT ‘Sebastian’ and *rhp1.e* mutant.

The *rhp1.e* mutant was isolated by the analysis of root hair phenotype of mutants carrying mutations in the *HvEXPB5* gene (*HORVU1Hr1G054230*) encoding type B expansin. These mutants were developed using TILLING strategy and the *Hor*TILLUS population created at the University of Silesia in Katowice in the background of the spring barley cv. ‘Sebastian’ [37]. One of them, named *hvexpb5.i*, showed extremely short root hairs inhibited at the stage of tip growth initiation (Figure 1A,B). Although expansins are involved in cell wall loosening that is required for root hair development [18,19,20], the abnormal root hair trait did not co-segregate with the C773T mutation detected in the *hvexpb5.i* allele (Supplementary Table S1). This indicated that the *hvexpb5.i* mutant carried an additional mutation responsible for the observed inhibition of root hair growth. We have previously found four allelic root hair mutants in barley with a very similar phenotype, named *rhp1* (*root hair primordium 1*), for the arrest of root hair growth at the primordial stage) (Supplementary Figure S1) [41]. Therefore, we decided to perform a complementation test between the *hvexpb5.i* and the *rhp1.b* mutant lines and we found that the mutants were allelic in regards to the root hair phenotype. The F_1_ hybrid plants of *hvexpb5.i* x *rhp1.b* cross and all analysed 150 F_2_ individuals exhibited the same extremely short root hairs as both parents. These data clearly showed that mutations responsible for the inhibition of root hair growth occurred in the same locus in both mutants. Thus, we assumed that the *hvexpb5.i* mutant carried another mutation, located in the *rhp1* gene, that caused its root hair phenotype. We named this mutation *rhp1.e*, as the next allele in the *rhp1* locus. Since the isolated mutant carried confirmed mutations in two genes, it should be designated *hvexpb5.i/rhp1.e*; however, for simplicity, we use only the *rhp1.e* name in the further parts of this paper. The described mutant was used for identification of the *rhp1* sequence and analysis of its function, which are presented in the further sections of this paper.

Detailed microscopic analysis of 7-day seedlings grown in aeroponic conditions revealed that plants carrying *rhp1.e* allele developed extremely short root hairs (64.3 ± 14.65 µm) in comparison to the WT cultivar ‘Sebastian’ (2147 ± 163.44 µm) (Figure 1A–C). The length of *rhp1.e* root hairs reached only about 3% of the root hairs length observed in the WT. The shape, morphology and density of root hairs in *rhp1.e* were comparable with the initial stage of root hair growth in the parent cultivar ‘Sebastian’. Additionally, we analysed the pattern of root epidermal cells in the elongation zone of *rhp1.e* mutant and its parent. We observed the similar percentage of the two types of cells in the root epidermis of both genotypes: the shorter ones that produced root hairs (trichoblasts) and the longer cells that did not develop root hairs (atrichoblasts) (Figure 1D,E).

Additionally, to determine whether the mutation responsible for the inhibition of root hair growth (or the additional mutation in the *HvEXPB5* gene carried by the *rhp1.e* mutant) affected other root traits, we examined the root system of the mutant and its parent. No differences in root system characters including the total length of roots, total volume and surface of root system between *rhp1.e* mutant and the WT ‘Sebastian’ were observed (Supplementary Figure S2).

### 2.2. Exome Sequencing Output Data

Taking into account the available data about *rhp1* gene location on barley chromosome 1H [41,43], we decided to use the whole exome sequencing (WES) approach, performed using the SeqCap EZ HyperCap workflow (Roche, Basel, Switzerland), to identify a candidate gene responsible for the mutant phenotype. To prepare library for DNA sequencing, we used DNA extracted from the leaf of the BC_1_F_2_ individual with the *rhp1.e* root hair phenotype and DNA of its WT parent ‘Sebastian’. Using whole exome sequencing, we obtained 95802780 and 112849935 raw reads for the mutant *rhp1.e* and the parent variety ‘Sebastian’, respectively. In both cases, the 99.2% of reads were mapped to the reference genome of cultivar ‘Morex’ (version Hv_IBSC_PGSB_v2; EnsemblPlants: https://plants.ensembl.org). Among these mapped reads, 59.3% and 60.2% aligned to unique regions, 33.99% and 38.84% mapped to duplicated regions on the reference genome for mutant and parent cultivar samples, respectively. The length of the aligned sequences ranged between 35 and 76 bp. The average coverage across target regions was 20.35 for the mutant *rhp1.e* sample and 24.89 for its parent cultivar, which suggests the good quality of samples sequencing.

Forty-six SNPs (single nucleotide polymorphisms) in a homozygous state were identified in the mutant sample in comparison to its parent variety using exome sequencing. The observed distribution of SNPs across the barley chromosomes was unequal and ranged from one SNP on chromosome 4H and 6H to 14 SNPs on chromosome 7H. All SNPs were found in different genes.

On the chromosome 1H, where a locus *rhp1* has been localized in our previous studies, eight mutations differing the *rhp1.e* mutant from its WT parent variety were identified (Table 1). Seven identified mutations were found to be missense, while one change was described as a splice junction mutation.

All eight genes carrying mutations identified by WES were evaluated as possible candidates for the *rhp1* locus. The previous data showed that our target gene is flanked by the SSR Bmag0382 and AFLP M49E44.MS301 markers (Supplementary Figure S3A). These markers could not be used to physically anchor the region of interest in barley genome. To achieve this goal, all available barley genetic maps were screened and the *HVA1* (*H. vulgare ABUNDANT PROTEIN*) gene was identified as a gene mapped nearby the Bmag0382 marker [44] (Supplementary Figure S3B). Using the barley genome sequence (version Hv_IBSC_PGSB_v2; EnsemblPlants), the *HVA1* gene (*HORVU1Hr1G079290*, designated *LEA3* in EnsemblPlants), was physically anchored between 522615373 bp and 522616624 bp on the barley chromosome 1H. Considering the physical localization of eight candidate genes with mutations within chromosome 1H, their ontology and potential impact of identified mutation on the encoded protein function, the *HORVU1Hr1G077230* was analyzed as the first candidate responsible for the *rhp1.e* root hair phenotype. This gene was the only candidate, except for *HvEXPB5*, annotated with biological functions associated with cell wall modification (Table 1). Additionally, the exome sequencing of the allelic mutant *rhp1.b* and its parent cultivar ‘Dema’ showed the presence of another mutation (T1827C substitution) only in the *HORVU1Hr1G077230* gene and the lack of mutations in the other seven candidate genes from the analyzed region (data not shown).

Analysis of 176 F_2_ individuals derived from the *rhp1.e* x ‘Sebastian’ cross revealed that all plants exhibiting the mutant phenotype carried the identified G1674A mutation in the homozygous state. Among F_2_ individuals that developed normal root hairs, the ratio of 2:1 for plants heterozygous for mutation to homozygous for WT allele was observed. There were no recombinants with the mutant root hair phenotype and the WT allele with G at 1674 position. These analyses supported the hypothesis that the identified G1674A mutation in the *HORVU1Hr1G077230* gene may be the cause of the *rhp1.e* mutant phenotype characterized by the extremely short root hairs.

### 2.3. Characterization of the HORVU1Hr1G077230 Gene and an Encoded Protein

**Figure 2:**
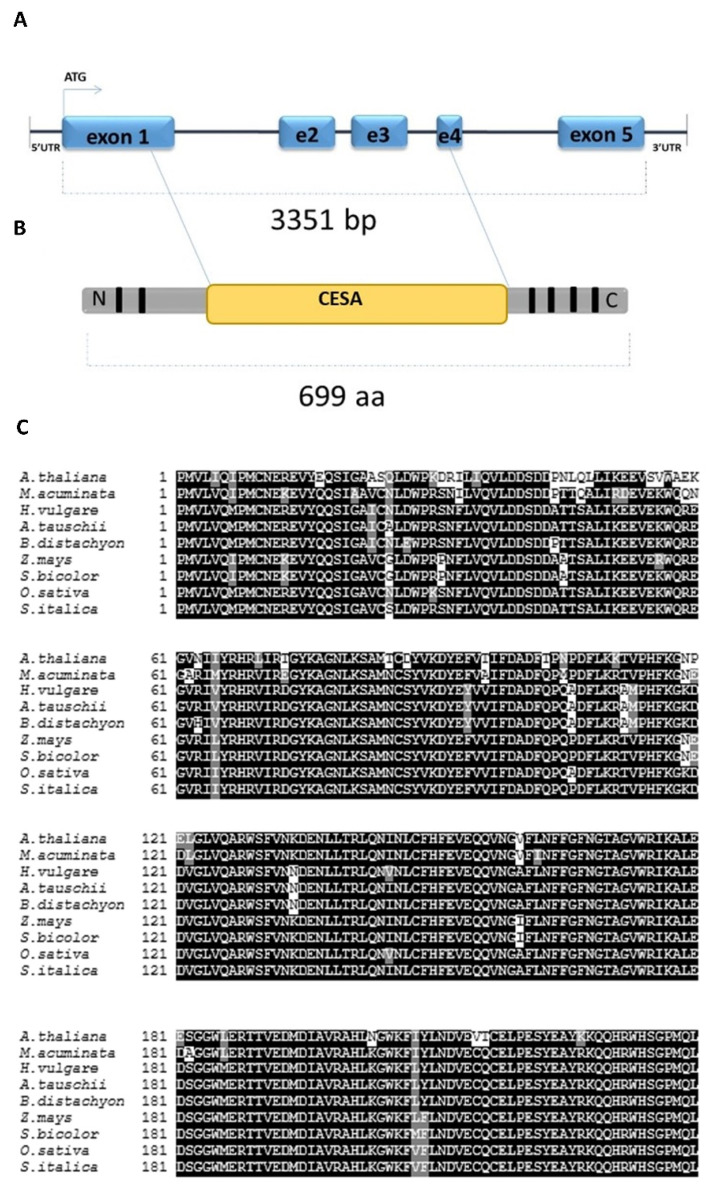
(**A**) The structure of *HvCSLC1* gene and (**B**) the scheme of HvCSLC1 protein. Blue boxes and lines in (**A**) represent exons and introns, respectively. Black boxes in (**B**) indicate the location of transmembrane domains (TDMs), blue—the cellulose synthase domain CESA. (**C**) The alignment of the CESA domain of HvCSLC1 protein sequence in *H. vulgare*, *Arabidopsis thaliana*, *Musa acuminata*, *Aegilops tauschii*, *Brachypodium distachyon*, *Z. mays*, *Sorghum bicolor*, *O. sativa* and *Setaria italica*. The black regions indicate the highly conserved amino acid residues.

Searching of the NCBI databases revealed that the *HORVU1Hr1G077230* gene encodes a cellulose synthase-like C1 (HvCSLC1) protein belonging to the CELLULOSE SYNTHASE-LIKE C (CSLC) family. Sequence analysis of the *HvCSLC1* showed that this gene is composed of five exons and the length of the genomic sequence from the start to the stop codon is 3351 bp (Figure 2A). The HvCSLC1 protein is 699 amino-acid long and possesses a CESA conserved domain that is located between 238 aa and 474 aa. Additionally, two transmembrane domains (TMDs) at NH_2_-terminus and four TMDs at the COOH-terminus were found within this polypeptide sequence (Figure 2B). The HvCSLC1 protein contains also a central catalytic D,D,D,QQHRW motif that was found in all CSLC proteins in rice, sorghum, poplar, grapevine and moss [45]. The multiple alignment indicates that *HvCSLC1* is an ortholog of the *OsCSLC7* (PLAZA 3.0; gene ID OS05G43530) of rice and of the *AtCSLC12* (PLAZA 3.0, gene ID AT4G07960) of Arabidopsis (Figure 2C, Supplementary Figure S4). In silico prediction showed that the HvCSLC1 protein is located in a Golgi apparatus and is probably necessary for XyG synthesis.

### 2.4. Analysis of the Identified Mutation in HvCSLC1

**Figure 3:**
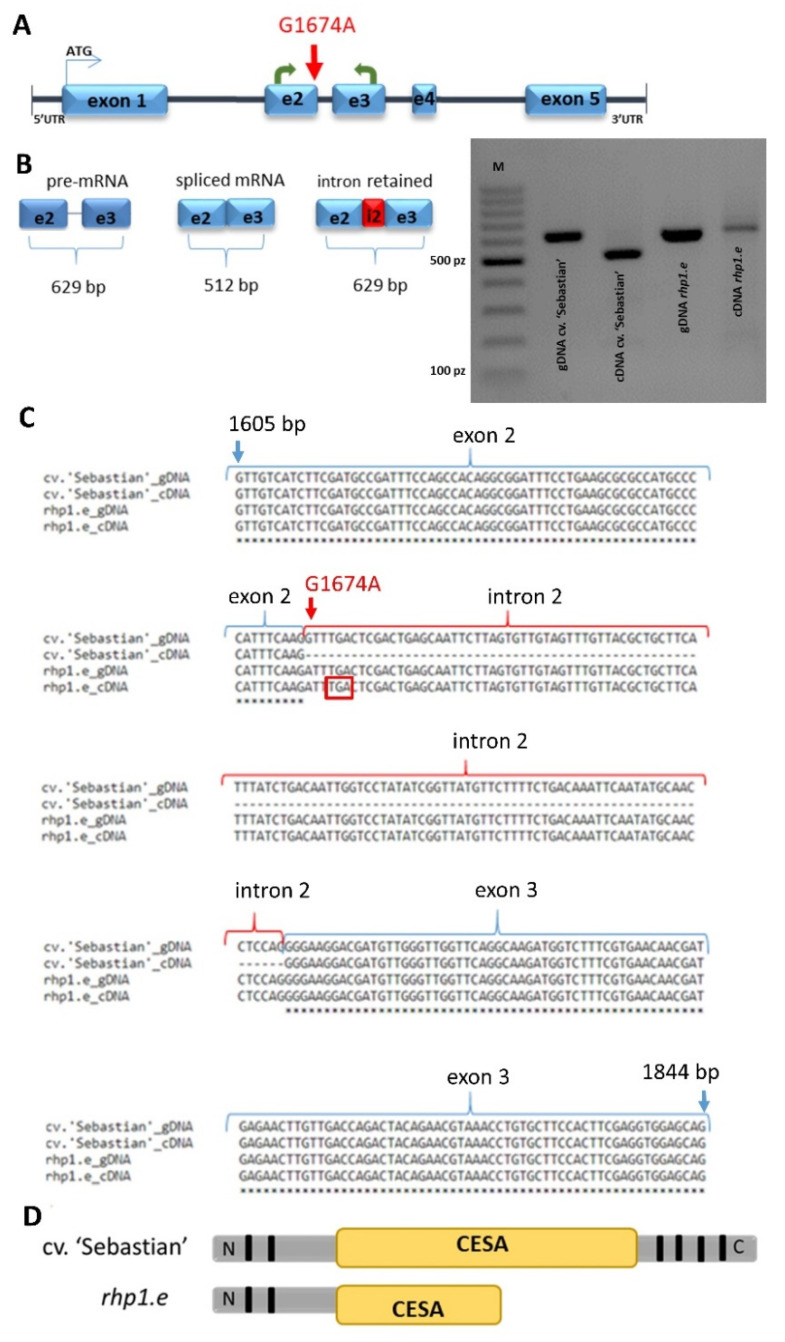
(**A**) The position of identified splice junction mutation in *HvCSLC1* gene (red arrow). Green arrows indicate the location of primes used in PCR amplification of a region flanking the mutation. (**B**) PCR products spanning the part of exons 2 and 3 of *HvCSLC1* sequence that were amplified using genomic DNA (gDNA) and cDNA as templates in cv. ‘Sebastian’ and *rhp1.e* mutant. (**C**) The alignment of a part of the *HvCSLC1* genomic (1605 bp to 1844 bp) and cDNA sequences representing fragment of exon 2, intron 2 and fragment of exon 3 of cv. ‘Sebastian’ and *rhp1.e* mutant. Red arrow indicates a splice junction mutation at the donor splicing site, red frame shows the premature STOP codon in the *rhp1.e* cDNA sequence. (**D**) The scheme represents the full length HvCSLC1 protein in the WT and the shorter protein with the lost part of amino acid sequence as a result of mutation in the *rhp1.e* mutant. Black boxes indicate location of transmembrane domains (TDMs); CESA—cellulose synthase domain.

The mutation identified in the *HORVU1Hr1G077230* gene of the *rhp1.e* was localized at the 516785752 bp position of chromosome 1H. It was a substitution of G to A nucleotide, located at the 1674 bp position from the transcription start site in the *HORVU1Hr1G077230* gene (Figure 3A).

This mutation was also confirmed using PCR method followed by Sanger sequencing. Analyses using bioinformatics tools revealed that the mutation was located at the 5′-splice site, known as a splice-junction donor site, between the second exon and the second intron and it caused the retention of the second intron in the HORVU1Hr1G077230 mRNA sequence of the *rhp1.e* mutant. To confirm this in silico prediction, the primers flanking this mutation and anchored in the second and third exon were designed, then the region spanning the G1674A substitution in the *HORVU1Hr1G077230* gene was amplified in the *rhp1.e* mutant and its and parent cultivar ‘Sebastian’ using genomic DNA and root cDNA as templates. This analysis showed a longer PCR product amplified on the *rhp1.e* cDNA template than in the WT parent, while the PCR fragments amplified on genomic template had the same size in both genotypes (Figure 3B). The cDNA amplification product was longer by 117 bp in the mutant which matched the length of the second intron. These data proved that the identified mutation caused the retention of the second intron and impacted the HORVU1Hr1G077230 mRNA structure. Further in silico analysis indicated that intron retention should result in a change of open reading frame (ORF) and occurrence of the premature stop codon UGA at the 4 bp position of the retained intron in the mRNA (Figure 3C). The premature stop codon is foreseen to cause the shortening of the predicted protein by 344 amino acids at the C-terminal site. As the whole HvCSLC1 protein is 699 aa long, the identified G1674A substitution in the encoding gene in *rhp1.e* mutant causes the loss of almost a half of the protein sequence including part of the CESA domain and C-terminal transmembrane domains (Figure 3D).

### 2.5. Identification of the Mutations in Allelic Mutants and Their Impact on the Protein Function

**Figure 4:**
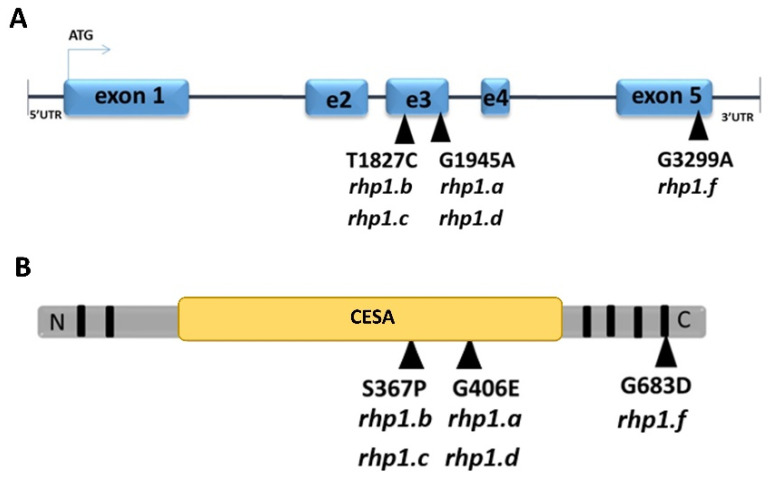
The location of identified mutations (black triangles) in (**A**) genomic and (**B**) protein sequence of *HvCSLC1* in allelic mutants *rhp1.a*, *rhp1.b*, *rhp1.c*, *rhp1.d* and *rhp1.f*. Black boxes indicate location of transmembrane domains (TDMs); CESA—cellulose synthase domain.

Analysis of the *HORVU1Hr1G077230* sequence in four *rhp1* mutants described previously [41] revealed that all of them carried a missense mutation in the gene sequence. Two different alleles in *HvCSLC1* gene were identified among *rhp1.a-rhp1.d* mutants. Mutants *rhp1.a* and *rhp1.d* carried the G1945A transition, that caused the change of glycine-406 to glutamic acid (G406E) in the protein sequence (Figure 4). In turn, *rhp1.b* and *rhp1.c* mutants carried the same one-base substitution, the T1827C transition, that caused the change of serine-367 to proline (S367P) in the protein sequence (Figure 4). Both alleles had mutations localized in the sequence encoding the conserved CESA domain of the HvCSLC1 protein. Positions of these mutations are conserved among plant species, from monocots to dicots. To predict the possible effect of the identified missense mutations on the protein function, the computational analysis using the PROVEAN tool was applied. This prediction showed that the change at the 367 position of serine (polar, neutral) to proline (nonpolar, neutral) (S367P) impacts the protein function with a score of −2.838. Considering the PROVEAN criteria, this mutation was recognized as deleterious. Similarly, the G406E change that caused the substitution of nonpolar, neutral glycine to polar, acidic glutamic acid was predicted to be damaging, with a score of −7.911.

Additionally, a similar root hair phenotype was observed in the *hvcbp20.ab* mutant identified previously in our TILLING population [46]. This mutant, in addition to the mutation in *HvCBP20* gene (that encodes a smaller subunit of cap-binding protein) carried an additional mutation responsible for the inhibition of root hairs at the initial stage. The root hair phenotype of the mutant segregated independently of the *hvcbp20.ab* mutation. We have crossed the *hvcbp20.ab* with the *rhp1.e* mutant and found that mutations responsible for root hair inhibition were allelic. Hence, a fourth, different allele (designated *rhp1.f*) was present in the *hvcbp20.ab* mutant. The proper name for this mutant line should be *hvcbp20.ab*/*rhp1.f*, but for simplicity, we use only the *rhp1.f* name in this article. Sequencing of the *HvCSLC1* gene revealed that *rhp1.f* allele possessed a substitution of nucleotide G3299 to A located within a fragment encoding a C-terminal one-helix transmembrane domain (Figure 4). This domain probably spans the cell membranes and is conserved among orthologs from rice to Arabidopsis. The PROVEAN prediction with a score of −5.206 clearly indicated that the change of glycine (nonpolar, neutral) to aspartic acid (polar, acidic) at the 683 position of the amino acid sequence is detrimental for the HvCSLC1 protein.

### 2.6. Expression Level of the HvCSLC1 and Other Genes Potentially Involved in the XyG Biosynthesis in Root Hairs

**Figure 5:**
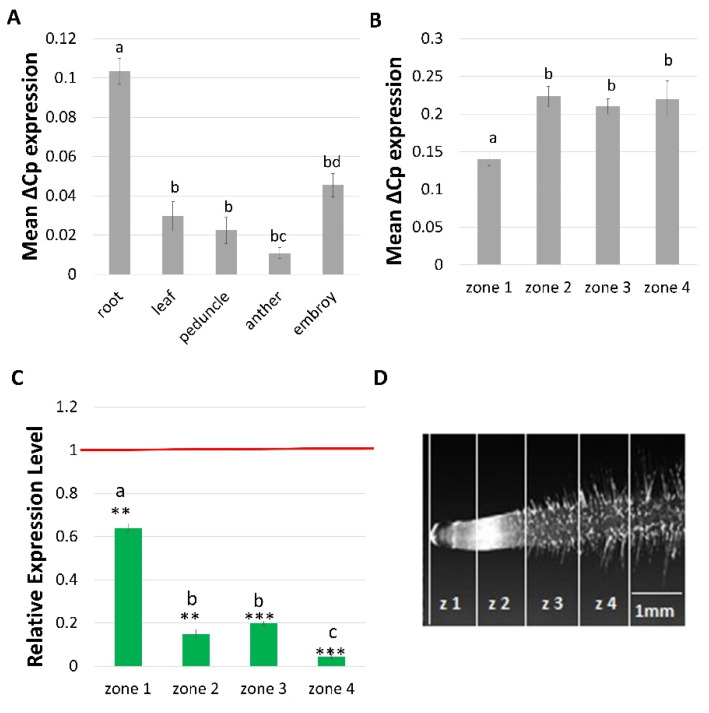
Expression profile of *HvCSLC1* gene in (**A**) various organs and (**B**) four root zones of WT cv. ‘Sebastian’. (**C**) Relative expression level of *HvCSLC1* in four root zones of *rhp1.e* mutant normalized to the gene expression in the corresponding zone of WT, which was considered as the value 1 (red line). Root zones: 1—meristematic, 2—elongation, 3—differentiation and 4—developed root hair zone. Different lowercase letters indicate the significant differences between analyzed organs (**A**) and root zones (B) of the WT ‘Sebastian’ and root zones of *rhp1.e* mutant (**C**) according to the One Way ANOVA, Tukey’s HSD test (*p* < 0.05). Asterisks indicate the significant differences between the mutant and WT parent according to the Student’s *t* test (*** *p* < 0.001; ** *p* < 0.01). Graph shows mean values of *n* = 3 with SEM. (**D**) Root zones that were used for analysis of gene expression (based on Kwasniewski et al., 2010 [47]).

The expression pattern of the *HvCSLC1* was analyzed in roots and the first leaf of 7-day-old seedlings and then in the peduncle, anther and mature embryo of the parent cultivar ‘Sebastian’. The transcripts of *HvCSLC1* were present in all examined organs; nevertheless, the highest expression level, normalized to the reference gene *ADP* (*ADP-RIBOSYLATION FACTOR 1*), was distinctly visible in the root (Figure 5A).

For studying the role of *HvCSLC1* in root hair morphogenesis, the transcript level of this gene was determined in the subsequent root zones of the *rhp1.e* mutant and its WT. The following four root zones were distinguished: (1) meristematic, (2) elongation, (3) differentiation and (4) developed root hair zone. One mm long root fragment of each zone was extracted from the WT and mutant roots and used for gene expression analysis. The expression of the *HvCSLC1* in the cv. ‘Sebastian’ was observed in all root zones, but the higher level was found in the root elongation zone and the successive sectors of root differentiation compared to the meristematic zone (Figure 5B). As the next step, we compared the *HvCSLC1* activity in different root zones of the *rhp1.e* mutant in relation to the *HvCSLC1* expression in its parent variety. The *HvCSLC1* transcript level was significantly lower in all root sectors of the *rhp1.e* compared to the corresponding zones of the WT. Interestingly, the *HvCSLC1* expression was reduced almost by half already in the meristematic zone of the mutant and decreased further in all the successive zones: elongation, differentiation and root hair zone (Figure 5C). The highest difference between the mutant and cv. ‘Sebastian’ was observed in the root hair zone (zone 4) where expression level of *HvCSLC1* was almost 25 times lower than in WT (Figure 5C).

**Figure 6:**
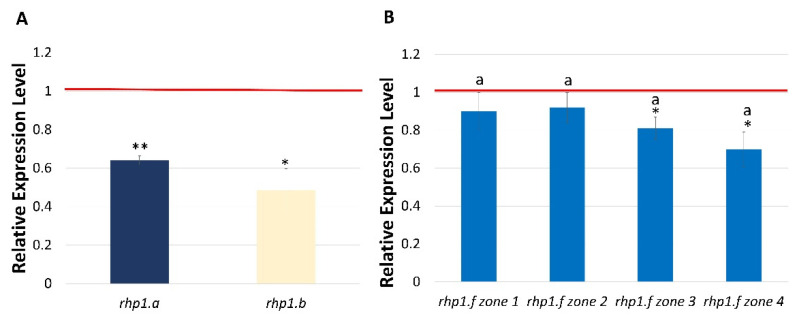
Expression profile of *HvCSLC1* gene in allelic *rhp1* mutants: (**A**) in roots of *rhp1.a* and *rhp1.b* and (**B**) in four root zones of *rhp1.f*, normalized to the expression level in the corresponding organs/root zones of WT, which was considered as the value 1 (red line). Root zones: 1—meristematic, 2—elongation, 3—differentiation and 4—developed root hair zone. Different lowercase letters indicate the significant differences between analyzed root zones of *rhp1.f* mutant according to the one way ANOVA, Tukey’s HSD test (*p* < 0.05). Asterisks indicate the significant differences between mutant and parent variety according to the Student’ *t*-test (** *p* < 0.01; * *p* < 0.05). Graphs show mean values of *n* = 3 with SEM.

Similarly, the decrease of *HvCSLC1* expression was observed in 1 cm root fragments of other allelic *rhp1* mutants in comparison to their parent varieties. Mutants *rhp1.a* and *rhp1.b*, carrying different amino acid substitutions in the CESA domain of the HvCSLC1 protein, showed a significant reduction of *HvCSLC1* expression in their roots (Figure 6A). Additionally, the expression of the *HvCSLC1* gene evaluated in the successive root hair zones of the *rhp1.f* mutant that carries an amino acid change in the C-terminal transmembrane domain (TMD), also showed a significant decrease compared to the WT parent, but only in the differentiation and root hair zones (Figure 6B).

**Figure 7:**
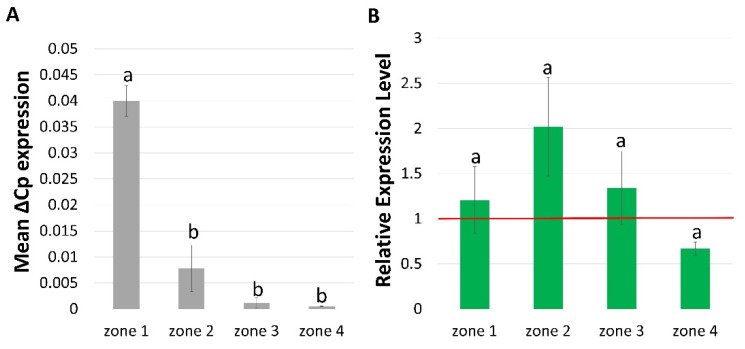
Expression profile of *HvCSLC3* gene in (**A**) four root zones of WT cv. ‘Sebastian’ (**B**) four root zones of *rhp1.e* mutant normalized to the gene expression level in the corresponding zones of WT, which was considered as the value 1 (red line). Root zones: 1—meristematic, 2—elongation, 3—differentiation and 4—developed root hair zone. Different lowercase letters indicate the significant differences between analyzed root zones in WT (**A**) and mutant (**B**) according to the one way ANOVA, Tukey’s HSD test (*p* < 0.05). Graph shows mean values of *n* = 3 with SEM.

To check whether *HvCSLC1* is the only cellulose synthase-like protein C involved in root hair tip growth, we examined the expression profile of *HvCSLC3* which encodes another XyG synthase active in barley roots [45]. Our studies revealed that the *HvCSLC3* transcript was significantly accumulated in the meristematic zone of the cultivar ‘Sebastian’ roots (Figure 7), contrary to *HvCSLC1*, which was expressed mainly in elongation, differentiation and mature root hair zones of the WT. Moreover, no difference in expression level of *HvCSLC3* was found in the *rhp1.e* mutant compared to the parent variety. It indicates that the *HvCSLC3* may be involved in XyG biosynthesis in a different part of the barley roots than the *HvCSLC1*.

The *HvCSLC1* gene encodes an enzyme predicted to control the first step of XyG synthesis, i.e., the formation of a XyG backbone [45]. To analyze if the mutation carried by the *rhp1.e* mutant in the *HvCSLC1* sequence may effect also further steps of XyG biosynthesis in elongating root hairs, we analyzed the expression of other genes which might be involved in the XyG biosynthesis and metabolism in barley. For this purpose, the seven candidate genes were selected based on literature data [23,45,48,49,50]. The predicted protein sequences encoded by the selected genes were applied for searching of putative orthologs in barley using PLAZA 3.0 and EnsemblPlants databases (Table 2). To date, the function of these genes in XyG biosynthesis have not been described in *H. vulgare*.

**Figure 8:**
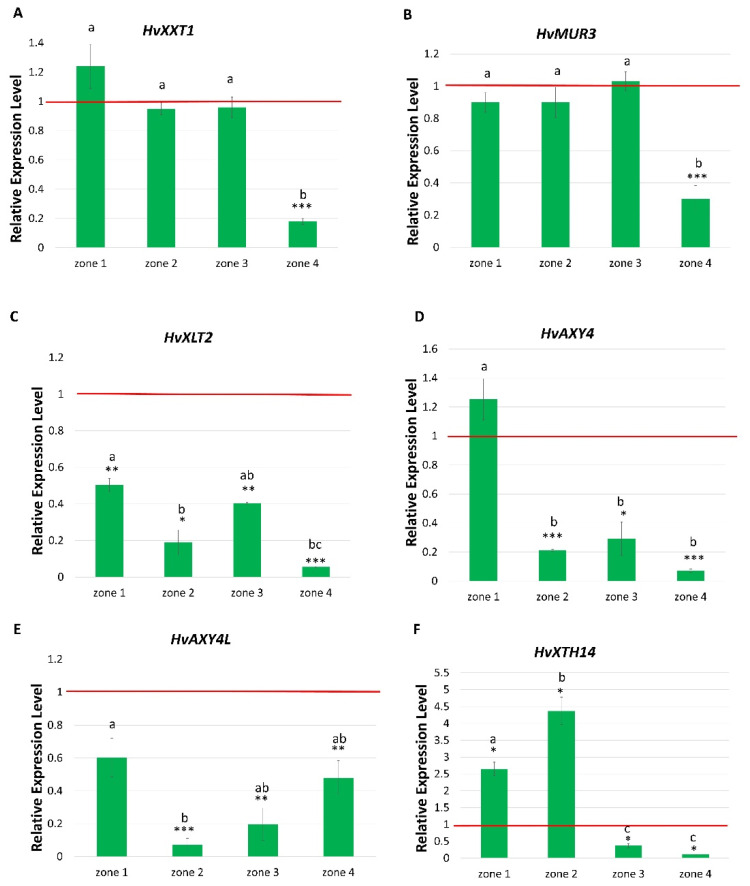
Expression profile of genes potentially involved in XyG synthesis in barley within four root zones of *rhp1.e* mutant. Relative expression level of each gene was normalized to the expression in the corresponding zones of the WT, which was considered as the value 1 (red line). (**A**) *HvXXT1*, (**B**) *HvMUR3*, (**C**) *HvXLT2*, (**D**) *HvAXY4*, (**E**) *HvAXY4L*, (**F**) *HvXTH14*. Root zones: 1—meristematic, 2—elongation, 3—differentiation and 4—developed root hair zone. Different lowercase letters indicate the significant differences between analyzed root zones in mutant according to the one way ANOVA, Tukey’s HSD test (*p* < 0.05). Asterisks indicate the significant differences between the mutant and parent variety according to the Student’s *t*-test (*** *p* < 0.001; ** *p* < 0.01; * *p* < 0.05). Graph shows mean values of *n* = 3 with SEM.

The first selected gene was *HvXXT1*, which was indicated as the ortholog of *OsXXT1* encoding a 6-xylosytransferase which links xylose to the β-glucan backbone in rice. In *rhp1.e* mutant, the expression of *HvXXT1* was decreased only in the mature root hair zone compared to the WT (Figure 8A). The similar expression pattern was noticed for *HvMUR3* (*H. vulgare MURUS3*), the ortholog of Arabidopsis *MUR3* gene encoding a xyloglucan-specific galacturonosyltransferase. Only in the mature root hair zone the transcript level of *HvMUR3* was significantly lower in *rhp1.e* mutant compared to the WT ‘Sebastian’ (Figure 8B). In Arabidopsis, the MUR3 contributes to xyloglucan side chain synthesis by adding the galactose on the third xylosyl unit within the XyG motif. In addition, a decreased level of expression of *HvXLT2* (*H. vulgare XYLOGLUCAN L-SIDE CHAIN GALACTOSYLTRANSFERASE POSITION 2*) encoding a xyloglucan galactosyltransferase that adds galactose to the xylosyl residue on the second position was observed in all root zones of *rhp1.e* mutant, with the lowest level in the mature root hair zone (Figure 8C). These results show that activity of all three genes, *HvXXT1, HvMUR3* and *HvXLT2*, responsible for linking xylose and next specific sugar residues to the xyloglucan backbone, was highly reduced in the mature roots hairs of *rhp1.e*. Interestingly, the expression of *HvMUR2* (*H. vulgare MURUS2*), the ortholog of Arabidopsis *MUR2* gene involved in fucosylation of galactose and galacturonic acid residues present at the XyG backbone, was not detected in barley, either in the WT or *rhp1.e* mutant.

The XyG backbone in Arabidopsis contains also acetyl groups. Two O-acetyltransferase, AXY4 (ALTERED XYLOGLUCAN 4) and AXY4L (ALTERED XYLOGLUCAN 4-LIKE) transfer acetyl groups specifically onto glucose residues within XyG chains in all major Arabidopsis tissues. In vitro analysis showed that acetylation can change the XyG features, resulting in the protection against enzymatic degradation [51]. Thus, we examined orthologs of two Arabidopsis genes: *AXY4* and *AXY4L* identified in the barley genome. Notably, the lower, compared to the WT, expression levels of *HvAXY4* and *HvAXY4L* were found in all root zones of *rhp1.e* mutant, except of *HvAXY4* in the meristematic zone (Figure 8D,E).

The next class of enzymes whose role in XyG modification has been proven comprises xyloglucan endotransglucosylases/hydrolases (XTH) involved in remodeling of XyG chains. In the Arabidopsis genome, 33 genes encoding XTH enzymes were identified and the role of the *XTH14* gene in root hair development has been already proven. The expression level of a barley ortholog, *HvXTH14*, was significantly lower in the differentiation and root hair zones of the *rhp1.e* root, compared to the corresponding zones of cv. ‘Sebastian’. Surprisingly, a significantly higher expression of *HvXTH14* was observed in meristematic and elongation root zones of *rhp1.e* than in the respective zones of the parent cultivar. This suggests the altered activity of the HvXTH14 enzyme in the mutant, although its role remains unclear (Figure 8F).

The presented results clearly show that the XyG synthesis pathway is disturbed in root hairs of the *rhp1.e* mutant. Furthermore, our data indicate that the activity of the *HvCSLC1* gene is specifically required for the tip growth of root hairs in barley. The proposed function of this gene is the synthesis of the XyG chains during root hair tube elongation.

## 3. Discussion

In the presented study, the *rhp1.e* mutant with extremely short root hairs was identified in the mutagenized TILLING population of spring barley (*H. vulgare* L.) cultivar ‘Sebastian’. The *rhp1.e* mutant showed root hair phenotype similar to some other root hair mutants (*rhp1.a-rhp1.d*) isolated and described in our previous studies [41,52]. In all these mutants, the growth of root hairs was arrested at the very early stage of root hair tube elongation. The genetic analysis revealed that this phenotype was caused by mutations occurring in the same gene localized on chromosome 1H [41]. To find the candidate gene for the *rhp1* locus, we created the F_2_ population of *rhp1.e* mutant x parent variety ‘Sebastian’ and performed the exome capture sequencing (called also whole exome sequencing, WES), a novel and efficient method for isolation of candidate gene sequences. The availability of high-quality genome assemblies for most crop plants, including barley, facilitated its application in studies of agronomic traits [53,54,55]. The NGS technology makes it possible to simultaneously define the region of interest and provide information on all genetic variations in the genome of the mutant line [55]. However, in species with large genomes, among them, barley, due to the large amount of sequencing data required to ensure adequate coverage of their genome, the complexity reduction strategies, such as exome capture and sequencing applied in this study, are required. It dramatically reduces sequencing costs while providing coverage for the vast majority of gene coding regions. Targeted exome sequencing in comparison to whole genome sequencing reduces barley genomic complexity more than 50-fold, dramatically reducing the sequencing and analysis load [56]. It should be noted that the exome sequencing approach has been successfully applied in barley, e.g., in the isolation of the *HvPHYC* (*H. vulgare PHYTOCHROME C*) gene encoding the photoreceptor protein PHYC involved in photoperiodic regulation of flowering time [57]. Similarly, the *Rht-B1* (*REDUCED HEIGHT B1*) gene that controls plant height in wheat was identified as a result of the whole exome sequencing of *T4-3822* mutant from the wheat EMS (ethyl methane sulfonate) population [55].

Using the WES approach, we have identified 46 SNPs between the *rhp1.e* mutant and its parent cultivar ‘Sebastian’, with eight of the identified SNPs present within chromosome 1H. The most likely causative mutation was found in the gene sequence of *HORVU1Hr1G077230* encoding a cellulose synthase-like C1 (HvCSLC1) probably involved in xyloglucan (XyG) synthesis [45]. Our analysis proved the impact of one base substitution G1674A, located in the second intron at the 5′ splice-junction site, on the splicing process of *HvCSLC1* pre-mRNA in the *rhp1.e* mutant. The retention of the second intron led to the presence of premature stop codon UGA and synthesis of truncated HvCSLC1 protein, shorter by 344 amino acids than its WT form. In silico prediction showed that mutated protein lacks the part of the highly conserved CESA domain with a central catalytic D,D,D,QQHRW motif involved in synthesis of the β-1,4-glucan chain [45]. Moreover, the mutation resulted in the loss of all four TMDs at the COOH-terminus which are involved in the transport of the glucan chain by forming the pores through Golgi membranes [58,59,60]. Furthermore, we detected three other mutations in the *HvCSLC1* gene that led to the inhibition of root hair elongation. All three mutations were missense changes with PROVEAN values indicating their deleterious impact on the protein function. None of these mutants carried a mutation in any of seven other candidate genes located on chromosome 1H which were indicated by WES in the *rhp1.e*. Thus, our findings provide strong evidence that mutations in the *HvCSLC1* gene cause the *rhp1* phenotype. We also demonstrated that sequencing of independent allelic mutants represents a powerful approach to identifying a target gene.

Based on Arabidopsis studies, it is known that the protein encoded by a *CELLULOSE SYNTHASE-LIKE C* (*CSLC*) gene is involved in the synthesis of the β-(1→4)-D-glucan backbone of xyloglucan [61]. XyG is the major hemicellulose of the primary cell wall (~20%) in dicots, while in grasses the primary wall comprises a relatively low (~5%) XyG content [62]. Interestingly, the role of XyG in maintaining cell wall structure has been proven in both clades [23,49]. XyG has a backbone of β-(1→4)-linked glucose residues, the same as a cellulose, but substituted with 1–6 linked xylose at the oxygen-6 position of the glucose units, in repeated pattern. The two types of xyloglucan pattern are distinguished: (1) XXGG-type characteristic for *Poaceae*, with two glucoses substituted with xyloses (XX) and two unsubstituted glucose residues (GG), and (2) XXXG-type, predominant in *Brassicaceae*, with three (of every four glucose residues substituted with a xylose residue) [63]. Additionally, the xylose may be capped with galactose (L), fucose (F/Z), galacturonic acid (Y) or acetyl groups, which depends on the cell type and plant family [64]. Most of the literature data indicate strong, non-covalent associations of xyloglucan with cellulose that assemble a molecular network, where XyG directly coats, tethers and separates the cellulose microfibrils. Thus, these interactions determine the cell wall flexibility during synthesis [65,66,67,68]. Biochemical studies revealed that XyG may bind selectively only to the specific surfaces on the cellulose microfibrils (biochemical hotspots) that are crucial for activity of cell wall relaxation proteins such as expansins. These studies revealed that the XyGs control rather the separation of cellulosic microfibrils at specific sites in the wall than the wall extensibility [62,69].

Five putative *CSLC* (*4,5,6,8,12*) genes were found in Arabidopsis, with two of them, *CSLC4* and *CSLC12*, highly expressed in root hairs [61]. However, a knock-out mutation of one gene only, *CSLC12*, caused a slight reduction of root hairs compared to the WT, while single T-DNA insertions in other *CSLC* genes had no effect on root hair growth [61]. The stronger effect on root hair length was observed in quadruple (*cslc4 cslc5 cslc6 cslc12*) and quinduple mutant lines (*cslc4 cslc5 cslc6 cslc8 cslc12*), indicating that *CSLC12* in cooperation with other *CSLC* genes are necessary for root hair growth [61]. In accordance with these results, single mutants containing a T-DNA in each of the five Arabidopsis *CSLC* genes had normal levels of XyG, reduced only in the higher order mutants [61]. In barley, five *CSLC* genes (*HvCSLC 1-5*) were identified [45]; however, the functional analysis of these genes have not been performed yet. Four of them (*HvCSLC 1-4*) have about 80% protein sequence identity. According to the phylogenetic analysis, *HvCSLC1* is an ortholog of the Arabidopsis *CSLC12* gene that encodes protein displaying 66.8% protein identity to HvCSLC1 (Supplementary Figure S4). Indeed, the ontology analysis of HvCSLC1 suggested the glucan synthase activity of this protein and its particular role in XyG biosynthesis. Additionally, our in silico prediction showed that HvCSLC1 is a Golgi apparatus protein and the synthesis of XyG is known to take place exclusively within the trans-Golgi cisternae [70,71,72]. Expression analysis conducted in our studies revealed the *HvCSLC1* activity in all examined organs (root, leaf, tiller, anther, embryo) of the WT cultivar, but the highest level of *HvCSLC1* transcripts was observed in roots. Furthermore, our results showed that a single mutation in the *HvCSLC1* sequence led to the similar root hair phenotype as the Arabidopsis higher-order *cslc* mutants. This finding suggests a different glucan synthase activity of barley *HvCSLC1* than *CSLC12* in Arabidopsis. It might be consistent with the various composition of the cell wall in monocots and dicots.

In the *rhp1.e* mutant with extremely short root hairs, the *HvCSLC1* expression level was highly reduced in all root zones (meristematic, elongation, differentiation and developed root hair zone) compared to the respective zones of the WT roots. The mutant carries a splice-junction mutation that results in a premature termination codon (PTC) in the retained second intron. The transcript with PTC might be a target for rapid degradation, presumably through a nonsense-mediated decay or translated into truncated protein [73]. The transcript levels of *HvCSLC1* in roots of three other allelic mutants (*rhp1.a, rhp1.b, rhp1.f*) carrying different missense mutations, were also significantly lower than in their respective parent cultivars; however, the mechanism leading to this decrease remain to be elucidated. Apart from the defect in root hair development, the mutants grew normally, without any visible differences in comparison to the WT.

The experiments performed by Dwivany and co-workers (2009) [45] showed that in addition to *HvCSLC1*, another gene, encoding cellulose synthase-like protein C, *HvCSLC3* (*H. vulgare CELLULOSE SYNTHASE-LIKE C3*), is highly expressed in the root tips of barley cultivars ‘Schooner’ and ‘Scoop’. Our studies confirmed the high expression level of *HvCSLC3* in the meristematic zone and showed its drastic decrease in other root zones of the WT cv. ‘Sebastian’, including the mature root hair zone. These data are opposite to the expression profile of *HvCSLC1* that was expressed mainly in elongation, differentiation and mature root hair zones. Moreover, no difference in expression level of *HvCSLC3* was found in the *rhp1.e* mutant compared to the parent variety. These results indicate that contrary to the *HvCSLC1*, the *HvCSLC3* is not specifically involved in XyG biosynthesis during barley root hair tip growth.

It is known that CSLC proteins cooperate with xylosyltransferase enzymes XXT that add the first side-chain xylosyl residues to the β-(1→4)-D-glucan backbone resulting in the synthesis of functional XyG [50,65,74,75,76,77]. In Arabidopsis, three xylosyltransferases (XXT1, XXT2, XXT5) are related to root hair elongation. The single *xxt5* mutant and the double *xxt1 xxt2* knockout mutant showed aberrant root hairs with bulged bases and slower growth in comparison to the WT. However, neither of the two single knockout mutants: *xxt1* or *xxt2* exhibited XyG deficiency phenotype [76]. Interestingly, a rice *OsXXT1* (*XYLOGLUCAN XYLOSYLTRANSFERASE 1*) may restore functional XyG structure and complement root hair defect when expressed in the Arabidopsis *xxt1 xxt2* double mutant, which suggests the same activity of *OsXXT1* as its Arabidopsis *XXT* homologs [23]. The rice mutant *srh1* (*short root hair 1*) carrying a mutation in the *OsXXT1* gene displayed extremely short root hairs in comparison to the WT, without differences in leaf and root growth. Authors suppose that the mutation of the *OsXXT1* gene causes the reduction of XyG content in the cell wall, which contributes to the weakening of its structure during root hair elongation [23]. We identified only one homolog of *OsXXT1* in the barley genome and its role in XyG biosynthesis has not been described yet. Our analysis showed a significant decrease of the *HvXXT1* expression in the root hair zone of *rhp1.e* mutant. Hence, we assume that addition of xyloses to β-glucan backbone may be disrupted during tip growth of the *rhp1* mutants. This finding is consistent with the studies of Arabidopsis *xxt1 xxt2* double mutant, in which the reduced expression l of xyloglucan xylosyltransferase genes resulted in the lower amount of xylose in root hair cell wall [76]. Taking into consideration that the expression level of *HvXXT1* gene has not been changed in the meristematic, elongation and differentiation zones of the *rhp1.e* mutant and that *HvCSLC3* gene had the similar expression profile, we assume that *HvXXT1* may cooperate with *HvCSLC3* in producing XyG in the root zones not containing fully developed root hairs.

Interestingly, the expression of *HvMUR3* gene, whose ortholog in Arabidopsis is involved in the further decoration on xyloses [35], was significantly lower in the root hair mature zone of the *rhp1.e* mutant in comparison to the WT parent. In Arabidopsis, the MUR3 belongs to the G47 (galactosyltransferases 47) protein family, similarly to XUT1 (XYLOGLUCANSPECIFIC GALACURONOSYLTRANSFERASE 1) and XLT2, and is responsible for attachment of the galactose residue on the third xylosyl unit within the XyG motif [35]. Taking into consideration that this type of XyG modification is not common in *Poaceae*, we suggest that *MUR3* may play a different role in barley than in Arabidopsis. Additionally, there is a lack of barley homolog of *AtXUT1* involved in biosynthesis of acidic XyG whose role in root hair elongation has been proven in Arabidopsis. Mutants with a loss-of-function of *AtXUT1* gene synthesize xyloglucan that lacks galacturonic acid and they exhibit shorter root hairs than the WT [35]. These findings suggest that acidic XyG plays a crucial role in normal expansion of a tip-growing root hair during its elongation. It is possible that *HvMUR3* may play the same function in barley as *XUT1* in Arabidopsis, i.e., it acts by adding a galacturonic acid residue to the xylose to form acidic XyG. This hypothesis is supported by studies conducted on tip-growing hyphae of moss *Physcomitrella patens*, where acidic XyG is present despite the lack of *XUT1* gene homolog [35].

The role of *XLT2* in addition of galactose residues to the second position of xyloglucan backbone has been already proven in dicots. The Arabidopsis double *xlt2 mur3* mutant synthesized a XyG fraction without galactose residues resulting in a dwarf phenotype. However, there are no data about defects in root hair development in this mutant [78]. Surprisingly, our analysis revealed the significantly lower expression level of *HvXLT2* gene, an ortholog of *AtXLT2*, in all root zones of the *rhp1.e* mutant in comparison to the WT. The high reduction of *HvXLT2* expression in all root zones of the *rhp1.e* indicates that its action in not specific to the elongation of root hair tubes. Another gene analyzed in our study was the ortholog of *MUR2* gene, which in Arabidopsis is involved in fucosylation of galactose and galacturonic acid residues present at the XyG backbone [32]. We identified the barley *HvMUR2* gene, but no expression was found in WT roots. Similarly, *OsMUR2* in rice was expressed at the very low level and could not be detected by RT-qPCR [79]. These findings suggest that the fucosylation of XyG structure is not a predominant modification in *Poaceae*.

Galactose residues in Arabidopsis XyG can be also decorated with the acetyl group. The O-acetylation of the polymer backbone impacts the XyG physiochemical properties such as conformation and hydrophobicity, while the O-acetylation of side chains is an alternative substitution that inhibits the enzymatic degradation of wall polysaccharides [51,80]. To date, two acetyltransferases, encoded by the *AXY4* gene and its paralog *AXY4L*, that mediate this modification have been identified in Arabidopsis [81]. Available transcription data showed that *AXY4* is expressed in all major Arabidopsis tissues, except for seeds, where the *AXY4L* gene takes its role [82]. However, the lack of acetyl group on the XyG backbone in Arabidopsis had no effect on plant growth [83]. In plants of the *Poaceae* family, the O-acetylation appears on unsubstituted glucose residues within the β-glucan backbone. We observed a decreased expression level of *HvAXY4* and *HvAXY4L* genes in the elongation, differentiation and mature root hair zone of the *rhp1.e* root, and *HvAXY4L* also in root meristem. Taking into account these data, we assume that similarly to *HvXLT2*, also *AXY4* and *AXY4L* are not related specifically to the elongation of root hair tube, but are associated with other processes in barley root cells. In grasses, contrary to Arabidopsis, the O-acetyl-substituents are present on majority of wall polymers (hemicelluloses, pectic polysaccharides and lignin) and the wall glycoproteins, except of cellulose [80]. Moreover, the degree of O-acetylation varies between species, developmental stages and cell types [84].

Extension of cell wall requires also the activity of XTH (ENDOTRANSGLUCOSYLASE/ENDOHYDROLASES) enzymes that take part in remodeling of XyG network by breaking and relegating XyG crosslinks during cell expansion. Our data showed the significant reduction of the *HvXTH14* transcript level in differentiation and root hair zone of the *rhp1.e* root compared to the WT. Contrary to the *HvXTH14* activity, the expression of genes not involved in XyG biosynthesis, such as *HvEXPB5*, has not been reduced in the root of the *rhp1.e* mutant compared to the WT (data not shown).

**Figure 9:**
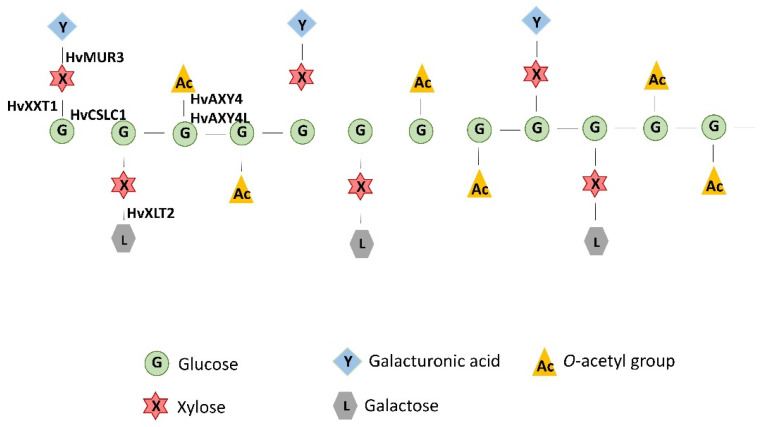
The scheme of XyG type occurring in barley root hair during tube elongation and the proteins potentially involved in its synthesis. HvCSLC1—cellulose synthase-like C1, HvXXT1—xyloglucan 6-xylosyltransferase, HvMUR3, HvXLT2—galactosyltransferases, HvAXY4—altered xyloglucan 4, HvAXY4L—altered xyloglucan 4-like.

Summarising our results, we assume that the mutation in the *HvCSLC1* might influence the pathway of XyG biosynthesis in the *rhp1.e* mutant during root hair elongation. Below, we present the model of XyG biosynthesis in barley roots which allows one to select new candidate genes for further functional analysis (Figure 9).

## 4. Materials and Methods

### 4.1. Plant Material

The *rhp1.e* mutant used in the exome capture experiment and another mutant with a similar phenotype, designated *rhp1.f*, were identified in the TILLING population developed at the University of Silesia in Katowice after double treatment of the spring barley cv. ‘Sebastian’ with sodium azide and N-methyl-N-nitroso urea (MNU) [37,46]. Four other mutants with identical root hair characteristics (root hairs inhibited at the primordial stage, designated *rhp1.a-d*), were isolated during our previous studies, after MNU mutagenesis of the spring cultivar ‘Dema’ (Supplementary Figure S1) [41,51]. All *rhp1.a-d* mutants are allelic and their root hair phenotype is controlled by a single recessive gene located on barley chromosome 1H [41,43]. The isolation of *rhp1.e* and *rhp1.f* mutants is described in the first part of the Results—Section 2.1.

For the exome capture experiment, *rhp1.e* mutant was back-crossed to the parent cv. ‘Sebastian’ and a BC_1_F_2_ line with mutant phenotype was used for DNA extraction and WES analysis, together with a DNA sample extracted from the ‘Sebastian’ cultivar. For confirmation of the causative mutation in the candidate gene, DNA samples from 156 F_2_ plants of *rhp1.e* x ‘Sebastian’ cross were used in the co-segregation analysis.

### 4.2. Growth Conditions and Root Hair Phenotyping

Seeds of mutants, their WT parent cultivars and F_2_ progeny of crosses *rhp1.e* x *rhp1.b* and *rhp1.e* x cv. ‘Sebastian’ were surface sterilized with 2.5% NaClO (Sigma-Aldrich, St. Luis, MO, USA) for 20 min and after thorough washing three times in sterile water, were placed in sterile plastic Petri dishes filled with wet filter paper. Seeds were incubated in darkness at 4 °C overnight and then transferred to a growth chamber at 18 °C for 24 h.

For root hair phenotyping, seedlings were grown in aeroponic conditions. Pre-germinated seeds were placed on the cotton bung between two sterile tubes sealed together with a parafilm, with the bottom tube covered by aluminum foil. The seedlings were grown for 7 days in a growth chamber under controlled conditions: photoperiod 16/8 h, temperature 18 °C and light intensity 180 μE/m^−2^s^−1^.

For phenotyping of the whole root system of *rhp1.e* mutant and its parent cv. ‘Sebastian’, germinated seeds were transplanted into acrylic tubes filled with sterile soda-lime glass beads. The experiment was conducted in the greenhouse for 14 days according to the protocol described by Slota et al. (2016) [85]. Then, the roots of the 14-day old seedlings were scanned and analyzed using the WinRHIZO software (Regent Instruments, Quebec, Canada). The total length of roots, root volume and surface area of roots in the mutant and WT were measured and compared using Student’s *t*-test (*p* < 0.05). The experiment was performed with four biological replicates, where each replicate represented six plants per genotype.

### 4.3. Microscopic Analysis

#### 4.3.1. Light Microscopy and the Scanning Electron Microscope (SEM)

The root hairs zone of 7-day old seedlings were observed by a light microscopy using the Stemi 2000-C (Carl Zeiss, Jena, Germany) stereoscopic microscope and AxioVision LE (Carl Zeiss, Jena, Germany) software. To confirm the root hair phenotype observed using light microscopy, the scanning electron microscope (SEM) was applied. Detailed analysis using SEM allowed to measure the length of mutant and WT root hairs. The 1 cm long fragment of root including root hair zone of 7-day old seedlings that had been grown in aeroponic conditions were collected and immersed in a 3% glutaraldehyde in a 0.1 M sodium phosphate buffer (pH 7.2) for 24 h at room temperature. After that, the roots were washed three times with the same buffer for 15 min each and next, post-fixed in a 2% osmium tetroxide in a phosphate buffer for 2 h at room temperature. Then, the roots were washed in the buffer three times for 15 min and dehydrated through an ethyl alcohol series (50%, 60%, 70%, 80%, 90%, 95% and 100%, 10 min at each step). The samples were dried in a Critical Point Pelco-CPD2 apparatus using carbon dioxide and then mounted on aluminum stubs with double-sided tape, sputter coated with gold in a Pelco SC-6 sputter coater and viewed and photographed using a FESEM S 4100 device (Hitachi High-Technologies Europe GmbH, Krefeld, Germany). Twenty root hair segments from mutant and WT were analyzed and photographed. The length of 500 root hairs was measured for each genotype and data were compared using the Student’s *t*-test (*p* < 0.05).

#### 4.3.2. Confocal Laser-Scanning Microscopy (CLSM)

For observation of epidermal cell types present in the root elongation zone of the WT and *rhp1.e* mutant, ten independent roots prior to root hair initiation (allowing analysis of >2000 epidermal cells) were collected for each genotypes. Autofluorescence of the cell wall was analysed by a confocal laser-scanning microscopy (CLSM, Zeiss LSM 510 META; Zeiss, Jena, Germany) with a 364 nm UV laser line equipped with a 375 nm band-pass filter.

### 4.4. DNA Isolation

Fragments of young seedlings leaves from all individual plants were placed in Silica Gel (POCH, Gliwice, Poland) and dried for seven days. After this time, the tissue was ground (60 s, 4 m/s in FastPrep^®^24, MP Biomedicals, Santa Ana, CA, USA) and total DNA was extracted according to a modified micro-CTAB method [86].

### 4.5. Genetic Analysis

The inheritance of the *rhp1.e* root hair phenotype was analyzed using F_1_ and F_2_ generations of the crosses between the *rhp1.e* mutant and its WT parent cv. ‘Sebastian’. Root hair phenotypes of 156 F_2_ plants were examined and segregation was confirmed by the χ^2^ 3:1 test. All plants used for genetic analysis were grown in aeroponic conditions described above. After seven days, the root hair zone was observed using light stereoscope microscopy and DNA was extracted from leaves for further analysis.

The F_1_ and F_2_ progeny of a cross between the *rhp1.e* and *rhp1.b* was tested to check the genetic relationship between mutations underlying their root hair phenotype. The root hair zone of 150 individuals of F_2_ generation were screened to confirm the allelic nature of the identified mutation.

### 4.6. Exome Capture Experiment Design and Candidate Gene Selection

The exome sequencing was performed using the SeqCap EZ HyperCap workflow (Roche, Basel, Switzerland). The exome capture probes were synthetized according to the SeqCap EZ Developer probe pool design 120426_Barley_BEC_D04.EZ, as described by Mascher et al. (2013) [56]. These probes cover 60 Mbp of a barley exome and annotate to approximately 40,000 genes [87]. For library preparation, the KAPA HyperPlus Library Preparation Kit workflow was applied (Roche, Basel, Switzerland). The 700 ng of genomic DNA was fragmented by enzyme mix (KAPA Frag Enzyme, Roche, Basel, Switzerland) into fragments, whose major fraction spanned the 180–250 bp size range. The following steps: end-repair and A-tailing, adapter ligation, dual-size selection and ligation-mediated-PCR steps were conducted according to manufacturer’s protocol (Roche, Basel, Switzerland). A final insert size of pre-capture library was targeted to be in the range 150–500 bp, with a peak at 320 bp. To determine the concentration, size distribution and check the quality of individual libraries, an Agilent Bioanalyzer DNA 1000 assay (Agilent Technologies, Santa Clara, CA, USA) was used. The barcoded libraries representing *rhp1.e* and ‘Sebastian’ samples were pooled together based on the specific index combinations recommended by the Illumina. Equal amounts of the libraries were used. The hybridization incubation of pooled libraries with exome capture probes was performed at 47 °C for approximately 18 h. The following steps: series of washes with buffers of decreasing stringency, captured library recovery, post-capture PCR and clean-up, were conducted according to the manufacturer’s guide (Roche, Basel, Switzerland). The size range of exome capture fragments were analyzed using the Agilent 2100 Bioanalyzer DNA 1000 Chip, the A260/A280 ratio (1.7–2.0) was analyzed on a NanoDrop1000 spectrophotometer (Thermo Fisher Scientific, Waltham, MA, USA) and the quantity of the library was examined using qPCR with KAPA Library Quantification Kit (Roche, Basel, Switzerland) according to the manufacturer protocol. Sequencing was performed for 2 × 75 paired-end sequencing according to standard protocols with an Illumina HiSeq (Illumina, San Diego, CA, USA) at the Medical University of Bialystok, Poland.

Whole exome sequencing data was analyzed according to the Genome Analysis Toolkit (GATK) recommendations [88]. Reads were trimmed using BBduk (https://sourceforge.net/projects/bbmap/, [access date: 26 March 2019]) and mapped to reference genome (version Hv_IBSC_PGSB_v2—EnsemblPlants, https://plants.ensembl.org/) using BWA [89]. Duplicates were removed using Picard (http://broadinstitute.github.io/picard/, [access date: 26 March 2019]). Variant calling was carried out using GATK HaplotypeCaller in GVCF mode. All samples were joint-genotyped using GATK GenotypeGVCFs. The discovered variants were hard filtered in accordance with the values recommended by GATK for WES (SNPs: QD < 2.0; MQ < 40.0; FS > 60.0; SOR > 3.0; MQRankSum < −12.5; ReadPosRankSum < −8.0; Indels: QD < 2.0; FS > 200.0; ReadPosRankSum < −20.0). Additionally, low quality genotypes (GQ < 40) and positions with very low coverage (DP < 5) were filtered out using an in house script. Identified variants were annotated using Variant Effect Predictor [90]. For subsequent analysis, only correctly oriented paired reads with properly insert size were selected.

In addition to WES of the *rhp1.e*, the similar exome capture analysis was performed for one of the allelic mutant, *rhp1.b* and its parent cultivar ‘Dema’, according to the procedure described above.

### 4.7. Identification of Mutations and Sequence Analysis of Selected Genes

The putative genomic, mRNA and amino acids sequences for analyzed genes were acquired from EnsemblPlants and NCBI (https://www.ncbi.nlm.nih.gov/) platforms. The identification of homologs and gene ontology analysis were carried out using PLAZA 3.0 and EnsemblPlants databases. Functional domains of the protein were determined using CDD (https://www.ncbi.nlm.nih.gov/Structure/cdd/cdd.shtml, [access date: 26 October 2020]). Identification of transmembrane domains was conducted with WoLF PSORT (http://wolfpsort.org/, [access date: 26 October 2020]; [91]). The sub-cellular location of a protein was predicted using ProtComp 9.0 (http://www.softberry.com/cgi-bin/programs/proloc/protcomppl.pl, [access date: 26 October 2020]). The multiple sequence alignment of orthologs was provided using the ClustalOmega tool. (http://www.ebi.ac.uk/Tools/msa/clustalo/, [access date: 5 November 2020]; [92]. The phylogenetic tree was constructed using the Phylogeny program (http://www.phylogeny.fr/, [access date: 4 October 2021]) with default settings [93].

The mutation identified in the candidate gene *HORVU1Hr1G077230* was confirmed by Sanger sequencing of the PCR-amplified gene fragment using genomic DNA and root cDNA of *rhp1.e* mutant and WT ‘Sebastian’ as templates. The whole genomic sequence of the candidate *HORVU1Hr1G077230* gene was also determined in allelic mutants *rhp1.a, rhp1.b, rhp1.c, rhp1.d* and *rhp1.f*. All primers used in PCR method were designed with the Primer3 program (http://bioinfo.ut.ee/primer3-0.4.0/, [access date: 15 January 2020]) (Supplementary Figure S5). Each 20 µL PCR sample contained a mixture of 9 µL ddH_2_O, 2 µL reaction buffer (1x), 1 µL dNTPs (5 mM), 2 µL forward primer (5 µM), 2 µL reverse primer (5 µM), 0,5 µL BSA (50 mg/mL), 0,5 µL Color Taq DNA polymerase (1U, EURx, Gdansk, Poland Poland) and 3 µL DNA (100 ng/µL). PCR amplification for all fragments was carried out under the following reaction conditions: initial denaturation for 3 min at 95 °C, 3 cycles of denaturation for 45 s at 95 °C, primer annealing for 20 s at a 62 °C and elongation for 1.2 min at 72 °C, 3 cycles of denaturation for 45 s at 95 °C, primer annealing for 20 s at a 60 °C and elongation for 1.2 min at 72 °C, 3 cycles of denaturation for 45 s at 95 °C, primer annealing for 20 s at a 58 °C and elongation for 1.2 min at 72 °C, 40 cycles of denaturation for 45 s at 95 °C, primer annealing for 20 s at a 56 °C and elongation for 1.2 min at 72 °C and final elongation for 5 min at 72 °C. Amplification of all PCR products were assayed using agarose gel electrophoresis and sequencing by the Sanger method.

The functional effect of mutations identified in the allelic mutants was predicted using the PROVEAN (http://provean.jcvi.org/, [access date: 10 March 2020]) tool [94]. PROVEAN is a Protein Variation Effect Analyzer which provides the impact of amino acid substitution or indel on the biological function of a protein based on sequence homology. This tool differentiates deleterious substitutions from the neutral ones using default cutoff at −2.5. The predicted variant is considered as a ‘deleterious’ when the PROVEAN score is equal to or below −2.5. If the PROVEAN score is above the threshold, the variant is described as a ‘neutral’.

### 4.8. RNA Isolation and Gene Expression Analysis

Plant tissues: roots, root segments (Figure 5D) and leaf from 7-days old seedlings, peduncles and anthers of 6-weeks old plants and embryos of the cv. ‘Sebastian’ and the *rhp1.e* mutant were homogenized in a sterile mortar containing 500 µL TriPure Isolation Reagent (SigmaAldrich, St. Luis, MO, USA). The total RNA was extracted using a RNAqueous™Total RNA Isolation Kit (Invitrogen, Waltham, MA, USA) protocol. The purity and yield of the extracted RNA was determined using UV-Vis NanoDrop^®^ ND-1000 (Thermo Fisher Scientific, Waltham, MA, USA). One μg of total RNA per sample was treated with RQ1 RNase-FreeDNase (Promega, Madison, WI, USA) and reverse transcription was performed according RevertAid First Strand cDNA Synthesis Kit (Thermo Fisher Scientific, Waltham, MA, USA) instructions. The obtained cDNA product was diluted 1:5 with water and 2 μL of this solution were added to reverse transcriptase-quantitative PCRs (RT-qPCR) master mix. All primers used in the RT-qPCR analysis were designed with the Primer3 program (http://bioinfo.ut.ee/primer3-0.4.0/, [access date: 15 January 2020]) (Supplementary Table S2). The RT-qPCR experiment was carried out in three biological repetitions and two technical repeats of each repetition using a LightCycler^®^480 SYBR Green I Master (Roche, Basel, Switzerland). Analyses were performed under the following reaction conditions: initial denaturation for 5 min at 95 °C, 42 cycles of denaturation for 10 s at 95 °C, primer annealing for 20 s at a temperature specific for the primers and elongation for 10 s at 72 °C. Denaturation for the melt curve analysis was conducted for 5 s at 95 °C, followed by 1 min at 65 °C and 98 °C with ramp of 0.1 °C/s for the fluorescence measurement.

The *ADP* gene (*ADP-RIBOSYLATION FACTOR 1*) characterized by constant expression pattern in all analyzed tissues was used as an internal control in the presented study, thus the relative expression levels of all genes were calculated and normalized to this reference gene using 2^(−ΔΔCt)^ method [95]. The fold change of gene expression in root zones of the *rhp1.e* mutant was normalized to the gene expression level observed in the parent variety ‘Sebastian’, which was considered as the value of 1. Statistical significance of gene expression differences between samples was calculated using the One Way (ANOVA) and Tukey’s HSD test (*p* < 0.05).

## 5. Conclusions

The *HvCSLC1* is the first gene whose function in elongation of root hairs has been confirmed in barley. We have found that *HvCSLC1* encodes a protein with glucan synthase activity that probably is involved in XyG biosynthesis in barley root hairs. Barley mutant with extremely short root hairs described in this study carries a splice-junction mutation in the *HvCSLC1* sequence that leads to the truncated protein lacking a part of a CESA conserved domain and four TMDs at the COOH-terminus. Whole exome capture analysis showed that the identified mutation is responsible for the inhibition of root hair elongation in the mutant. The link between the *HvCSLC1* gene and root hair phenotype was confirmed by three independent allelic mutations identified in different plants. Our data demonstrate that the glucan synthase activity of *HvCSLC1* is an important factor in cell walls during tip growth of root hair cells in barley, irrespectively of a relatively low XyG content in the cell wall in *Poaceae*. We predict that the HvCSLC1 enzyme might be involved in the synthesis of XyG backbone specifically during root hair tube elongation, providing the structural capacity and flexibility of the tip-growing root hair cell wall.

## Figures and Tables

**Table 1:** Mutations identified on chromosome 1H using whole exome sequencing (WES).

Mutation Position on Chromosome 1H (bp)	Nucleotide in Reference and WT	Altered Nucleotide	Type of Mutation Identified	Gene ID	Encoded Protein	Ontology	Ortholog ID in *A. thaliana* or *O. sativa*	Encoded Protein	Ontology
18663714	C	T	Missense	HORVU1Hr1G008530	RNA polymerase II transcription factor	Regulation of transcription	AT2G42780	Transcription elongation factor B polypeptide	Transcription elongation from RNA polymerase II promoter
243015321	G	A	Missense	HORVU1Hr1G036300	Unknown	Nucleic acid binding	AT3G04610	RNA-binding KH domain-containing protein/flowering locus KH domain (FLK)	Positive regulation of flower development
400988293	C	T	Missense	HORVU1Hr1G054230 (MLOC_43237)	Beta expansin protein 5 (HvEXPB5)	Cell wall loosening	AT1G65680	Beta expansin protein 2 (EXPB2)	Cell wall loosening
443922454	G	A	Missense	HORVU1Hr1G061210	Transcription termination factor MTERF4 (mitochondrial transcription termination factors 4)	Plastid rRNA transcription, response to oxidative stress	AT4G02990	Plastid-localizated protein homologous to mitochondrial transcription termination factors (mTERF) found in animal	Plastid rRNA transcription
445822069	G	A	Missense	HORVU1Hr1G061630	Serine protease HTRA1 (high-temperature requirement-A1)	Serine-type endopeptidase activity	No best ortholog found	-	-
455806946	G	A	Missense	HORVU1Hr1G063510	Unknown	Oxidoreductase activity	AT4G30720	FAD/NAD(P)-binding oxidoreductase family protein	Oxidation-reduction process
502083971	C	T	Missense	HORVU1Hr1G073230	Coronatine insensitive 1-like protein (COI1)	Regulation of jasmonic acid mediated signaling pathway	AT2G39940	Coronatine insensitive 1 protein (COI1)	Jasmonic acid mediated signaling pathway
516785752	G	A	Splice junction	HORVU1Hr1G077230	Synthase celullose-like protein C1 (HvCSLC1)	Transferase activity, transferring glycosyl groups	Os05g0510800	Cellulose synthase-like protein C7 (CSLC7)	Cell wall organization

**Table 2:** The candidate genes selected for expression analysis potentially involved in XyG biosynthesis in barley root hairs.

Species	Gene	Ensembl/TAIR ID	Encoded Protein	Function (UniProt)	Barley Ortholog	Ensembl ID
*Oryza sativa*	*OsXXT1*	Os03g0300000	Xyloglucan xylotransferase 1	Transfers an alpha-D-xylosyl residue from UDP-D-xylose to a glucose residue in xyloglucan, forms an alpha-(1->6)-D-xylosyl-D-glucose linkage	*HvXXT1*	HORVU4Hr1G054910
*Arabidopsis thaliana*	*XUT1 (RHS8)*	AT1G63450	Xyloglucan-specific galacturonosyltransferase (root hair specific 8)	Forms the beta-D-galactosyluronic acid-(1->2)-alpha-D-xylosyl linkage, required for root hair development probably by providing important acidic xyloglucans	*-*	No best ortholog found
*Arabidopsis thaliana*	*MUR3*	AT2G20370	Xyloglucan L-side chain galactosyltransferase position 3	Involved in the attachment of the Gal residue on the third xylosyl unit within the XXXG core structure of xyloglucan	*HvMUR3*	HORVU4Hr1G079030
*Arabidopsis thaliana*	*XLT2*	AT5G62220	Xyloglucan L-side chain galactosyltransferase position 2	Forms the beta-D-galactose-(1->2)-alpha-D-xylosyl linkage of xyloglucan	*HvXLT2*	HORVU0Hr1G005330
*Arabidopsis thaliana*	*MUR2*	AT2G03220	Galactoside 2-alpha-L-fucosyltransferase	Galactoside 2-alpha-L-fucosyltransferase activity, Is both necessary and sufficient for the addition of the terminal fucosyl residue on xyloglucan side chains, but is not involved in the fucosylation of other cell wall components	*HvMUR2*	HORVU6Hr1G078630
*Arabidopsis thaliana*	*AXY4 (TBL24)*	AT1G70230	Xyloglucan O-acetyltransferase 4	Transfers of an acetyl group onto O-6 of galactose or glucose residues	*HvAXY4*	HORVU7Hr1G039420
*Arabidopsis thaliana*	*AXY4L (TBL22)*	AT3G28150	Xyloglucan O-acetyltransferase 4-like	Transfers of an acetyl group onto O-6 of galactose or glucose residues	*HvAXY4L*	HORVU7Hr1G039180
*Arabidopsis thaliana*	*XTH14*	AT4G25820	Xyloglucan endotransglucosylase/hydrolase	Breaks a beta-(1->4) bond in the backbone of a xyloglucan and transfers the xyloglucanyl segment on to O-4 of the non-reducing terminal glucose residue of an acceptor, which can be a xyloglucan or an oligosaccharide of xyloglucan	*HvXTH14*	HORVU2Hr1G101240

## Data Availability

All material presented here is available upon request: iwona.szarejko@us.edu.pl. The Supplementary Materials are attached at the end of this file. Sequencing data from exome capture analysis are available at Sequence Read Archive (SRA; https://www.ncbi.nlm.nih.gov/sra) under the BioProject ID: PRJNA767208.

## Supplementary Materials

The following are available online at https://www.mdpi.com/article/10.3390/ijms222413411/s1.
