# Supplementary material for: Whole Exome Sequencing-Based Identification of a Novel Gene Involved in Root Hair Development in Barley (Hordeum vulgare L.)
Source: Int J Mol Sci. 2021 Dec 14;22(24):13411. doi: 10.3390/ijms222413411 (PMC8709170; doi:10.3390/ijms222413411)
Supplement: Supplementary file 1 [file ijms-22-13411-s001.zip › K Gajek_Suppl Mat_IJMS_13122021.pdf]

Table S1. Analysis of F_2 progeny of a cross *hvexpb5.i* x parent cv. 'Sebastian' (WT). The *hvexpb5.i* mutant carries C773T mutation in the *HvEXPB5* gene and exhibits extremely short root hairs.

Genotypic classes in regards to C773T mutation in the *HvEXPB5* gene	Number of F_2 plants with	
	Wild type root hairs	Short root hairs
Homozygous for mutation	13	20
Heterozygous for mutation	75	27
Homozygous for the WT allele	39	0

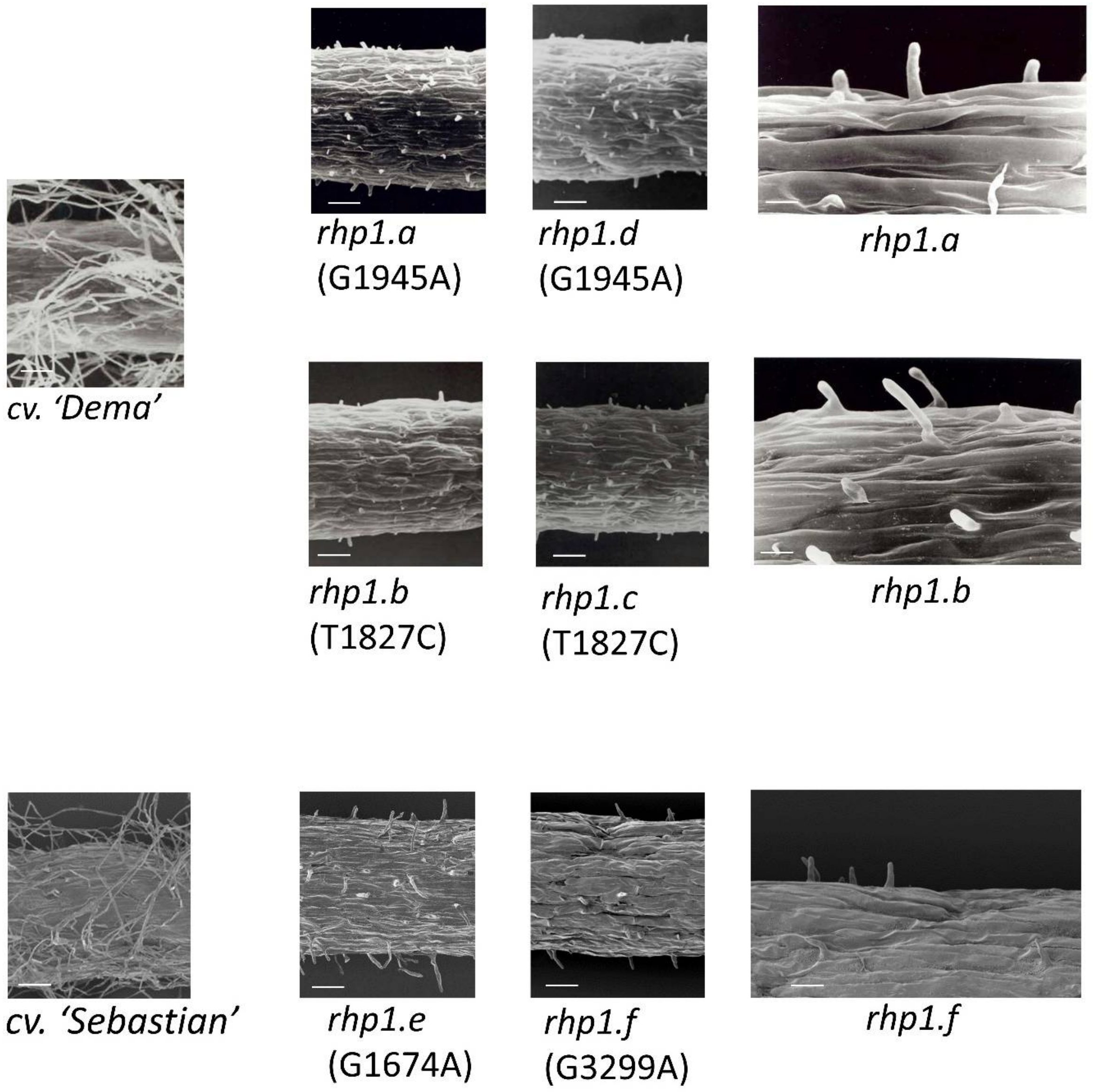

Figure S1. SEM images of barley *rhp* (*root hair primordia*) mutants and their parent varieties. Root hair zone of 5-7-old day old seedlings. Bars = 100 μm.

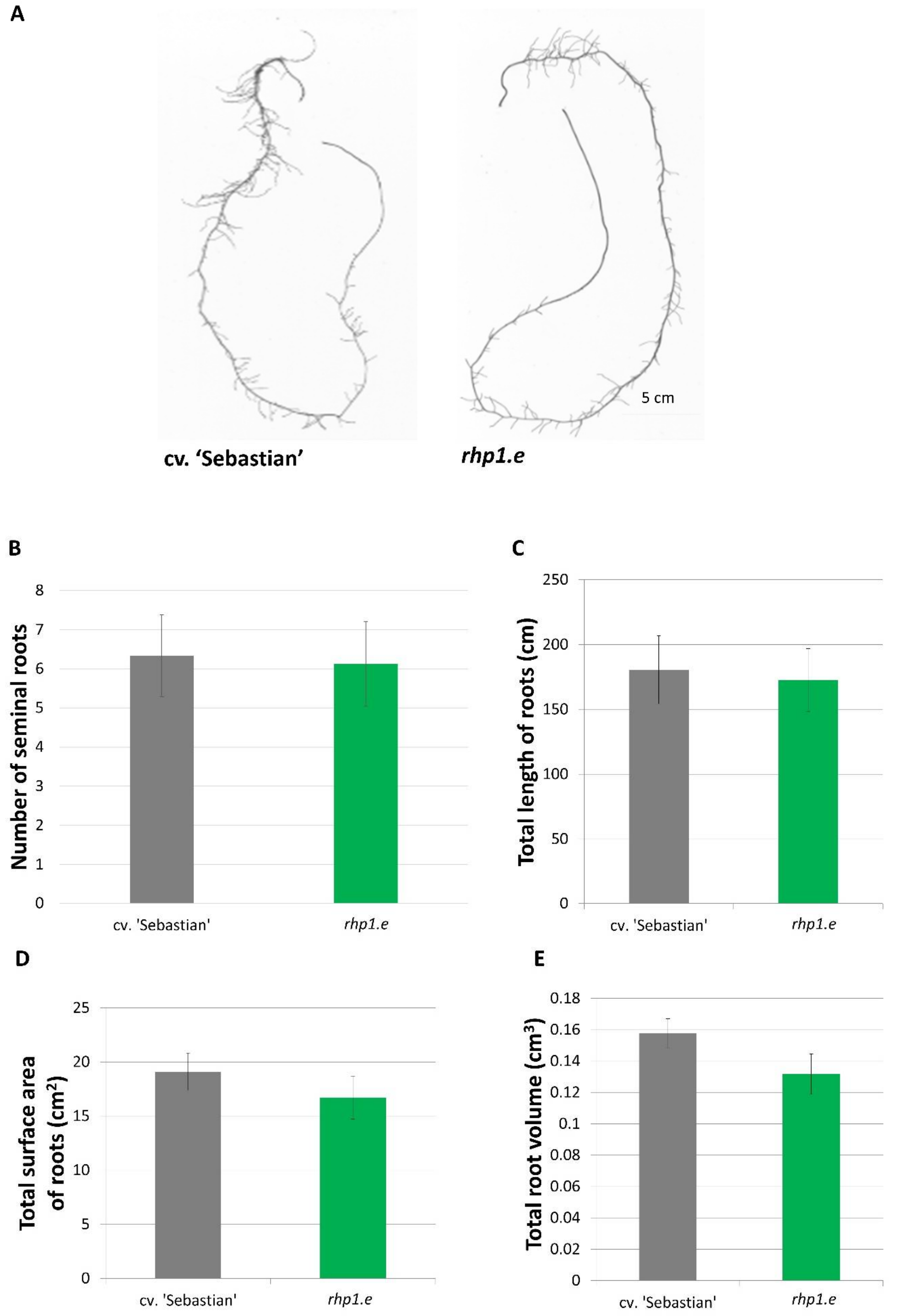

Figure S2. Root system analysis of *rhp1.e* mutant and WT cv. 'Sebastian' at 14-day seedling stage. **(A)** The longest seminal root in mutant and WT. Root system parameters of *rhp1.e* mutant and WT plants: **(B)** Number of seminal roots, **(C)** Total length of roots, **(D)** Total surface area of roots and **(E)** Total root volume. Significant differences between *rhp1.e* mutant and WT were estimated using Student's t-test. Graphs show mean value of four biological replicates (each replicate represented six plants per genotype) with SD.

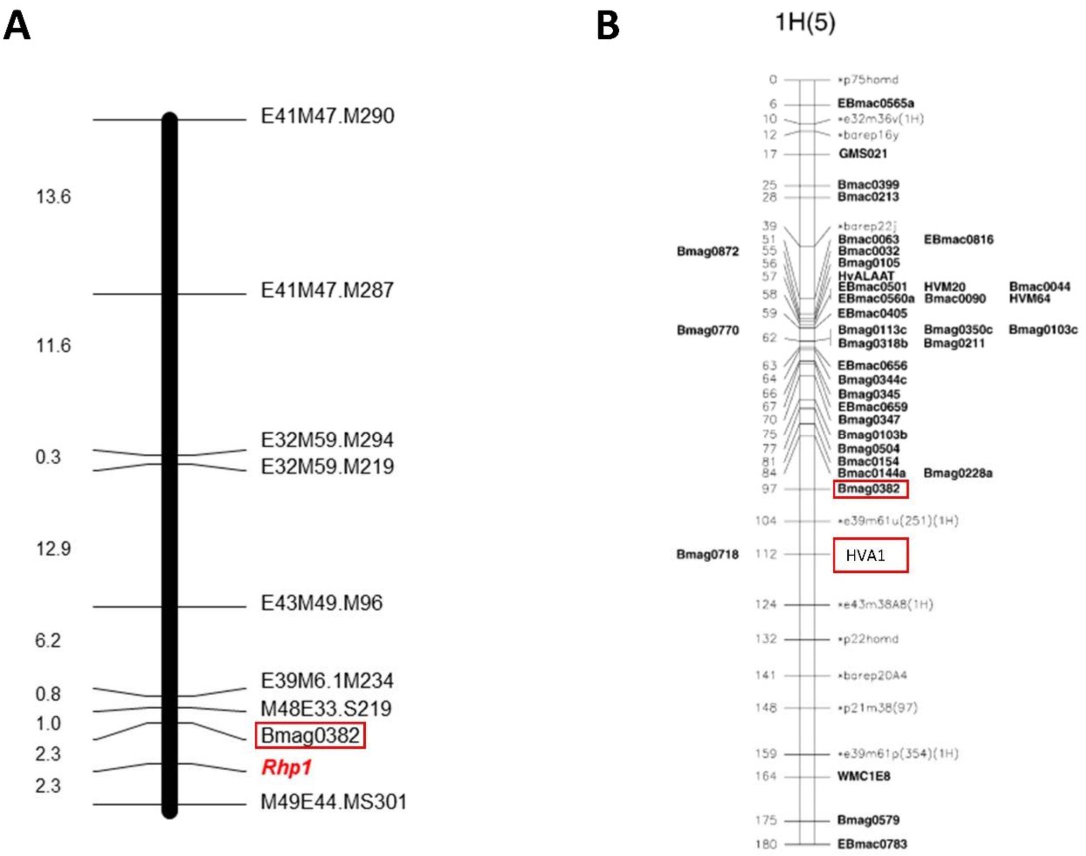

Figure S3. Location of *rhp1* gene on barley chromosome 1H. **(A)** Linkage group of *rhp1* gene on chromosome 1H based on the *rhp1.b* x 'Morex' mapping population (from Chmielewska et al., 2014 [41]), **(B)** Molecular linkage map of barley of 'Lina' x *Hordeum spontaneum* Canada Park population (based on Ramsay et al., 2000 [44]).

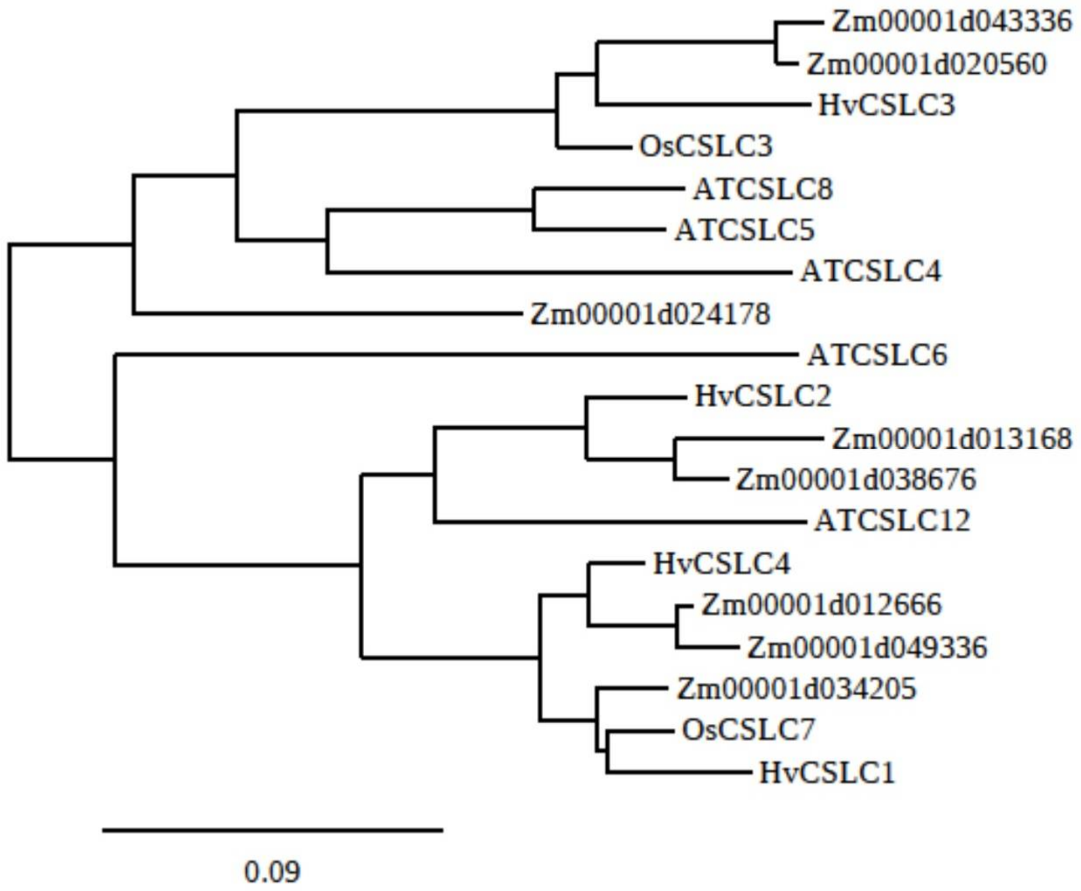

Figure S4. Phylogenetic analysis of CSLC members expressed in the root of *A. thaliana* (AT), *Z. mays* (Zm), *O. sativa* (Os) *and H. vulgare* (Hv). The tree was generated using Phylogeny.fr (www.phylogeny.fr) in "OneClick" mode [93]. This pipeline connects the following programs: MUSCLE for multiple alignment, Gblocks for automatic alignment curation, PhyML with the approximate Likelihood Ratio Test of branch support for tree building and TreeDyn for tree drawing.

A

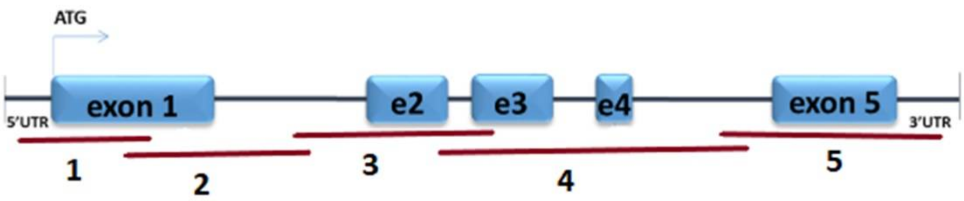

B

Primer sequence	Ta	Amplified fragment No.
Forward<br>CTCGTTTCGGTTTGACACG<br>Reverse<br>ATCTCGGGCATCTCAAGGT	56	1
Forward<br>GCTTCTACGGCTGCCTCA<br>Reverse<br>ACCGGTGACGACAAACTTCT	56	2
Forward<br>TCCAATCACGACTCTGATGC<br>Reverse<br>CGAAGTGGAAGCACAGGTTT	56	3
Forward<br>CAAGGTTTGACTCGACTGAGC<br>Reverse<br>AGATGTGCTGGCATGAATTG	56	4
Forward<br>CACCCTCTTATACTCAAGCAGTCA<br>Reverse<br>CCCGTCCAAAGGGTTTAGAT	56	5

Figure S5. (**A)** The structure of the candidate gene *HORVU1Hr1G077230* and fragments used for its amplification. (**B)** The list of primer sequences used for PCR amplification of the *HORVU1Hr1G077230* gene in *rhp1* mutants. Ta - annealing temperature

Table S2. The list of primer sequences used in qPCR analysis. Ta - annealing temperature

Gene	Primer sequence	Ta
HvCSLC1	Forward<br>ATCGTCCCGTACCTCCTCTT<br>Reverse<br>CTTCTTGGTGACCACCCACT	56
HvXXT1	Forward<br>GGTCGAGTTCTTCTGGTGGA<br>Reverse<br>TGTCGTCGTAGACCATCTCG	56
HvMUR3	Forward<br>AATCTGACTGGGGCAACAAC<br>Reverse<br>CTTTGGCAGGGTGGAAGTAA	56
HvXLT2	Forward<br>CTGCTCGCCATGTGTGATAC<br>Reverse<br>CGACAAGCTGGTCCCTTC	56
HvMUR2	Forward<br>GGCAACATGCTCAAGAACAA<br>Reverse<br>TCGTGCTCGAGGTAGACGTA	56
HvAXY4	Forward<br>CACCGTCTCCATCTTCTGGT<br>Reverse<br>TCGAGGAACACGTTGTTGTG	56
HvAXY4L	Forward<br>CTCTACTCGTCCCGGAGCTT<br>Reverse<br>GATCGTCGTCATCCTCTGGT	56
HvXTH14	Forward<br>ACACCAACGTGTATGCCAGA<br>Reverse<br>TGGGTCCAAATGATGCTGTA	56
HvCSLC3	Forward<br>ATGAGCTGCGACTACGTCAA<br>Reverse<br>AGGTACGGTGAGCTTGAGGA	56
