# Supplementary figures and images for: Whole Exome Sequencing-Based Identification of a Novel Gene Involved in Root Hair Development in Barley (Hordeum vulgare L.)
Source: Int J Mol Sci. 2021 Dec 14;22(24):13411. doi: 10.3390/ijms222413411 (PMC8709170; doi:10.3390/ijms222413411)

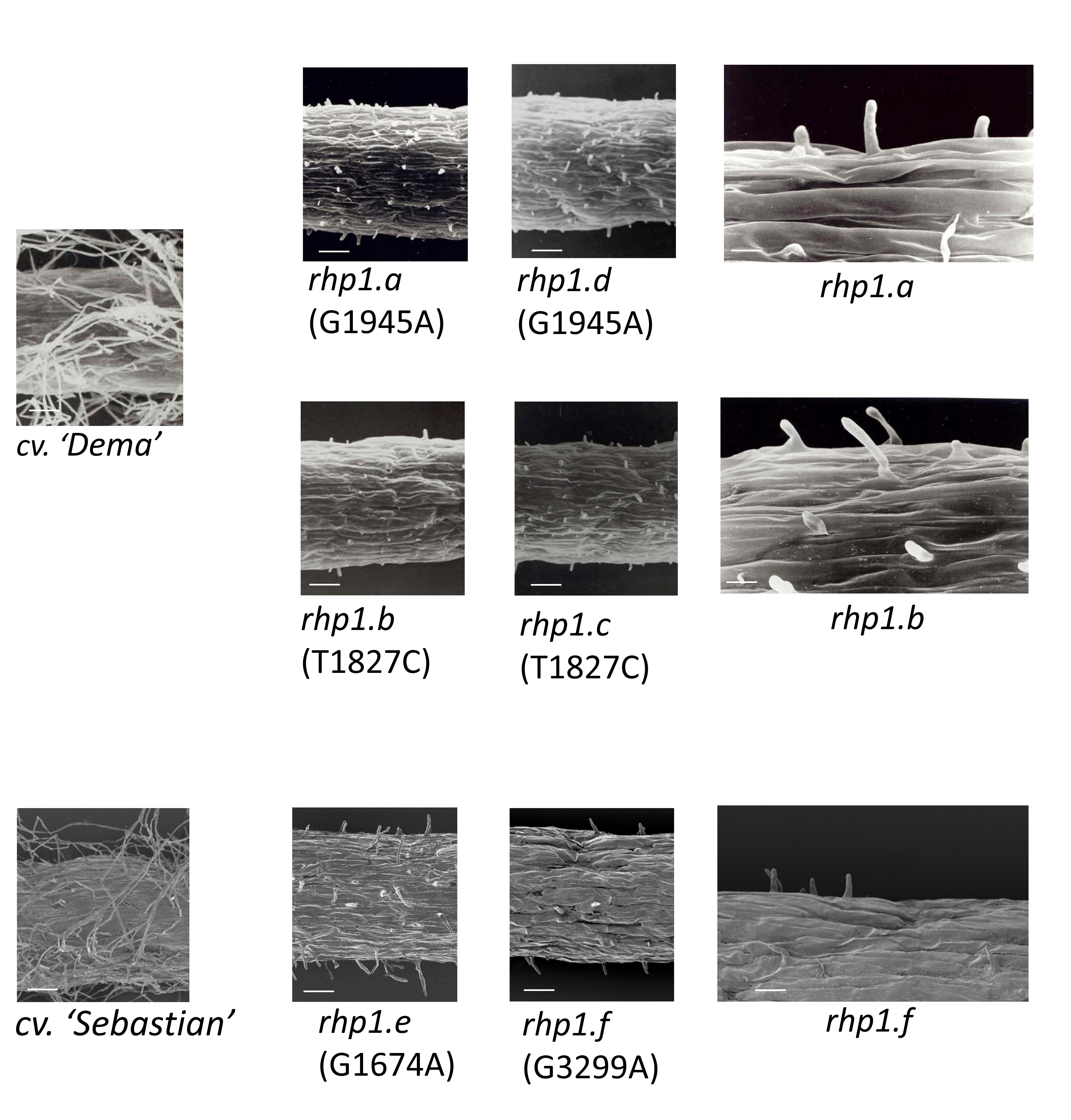

Supplement: Supplementary file 1 [file ijms-22-13411-s001.zip › SF_S1.jpg]

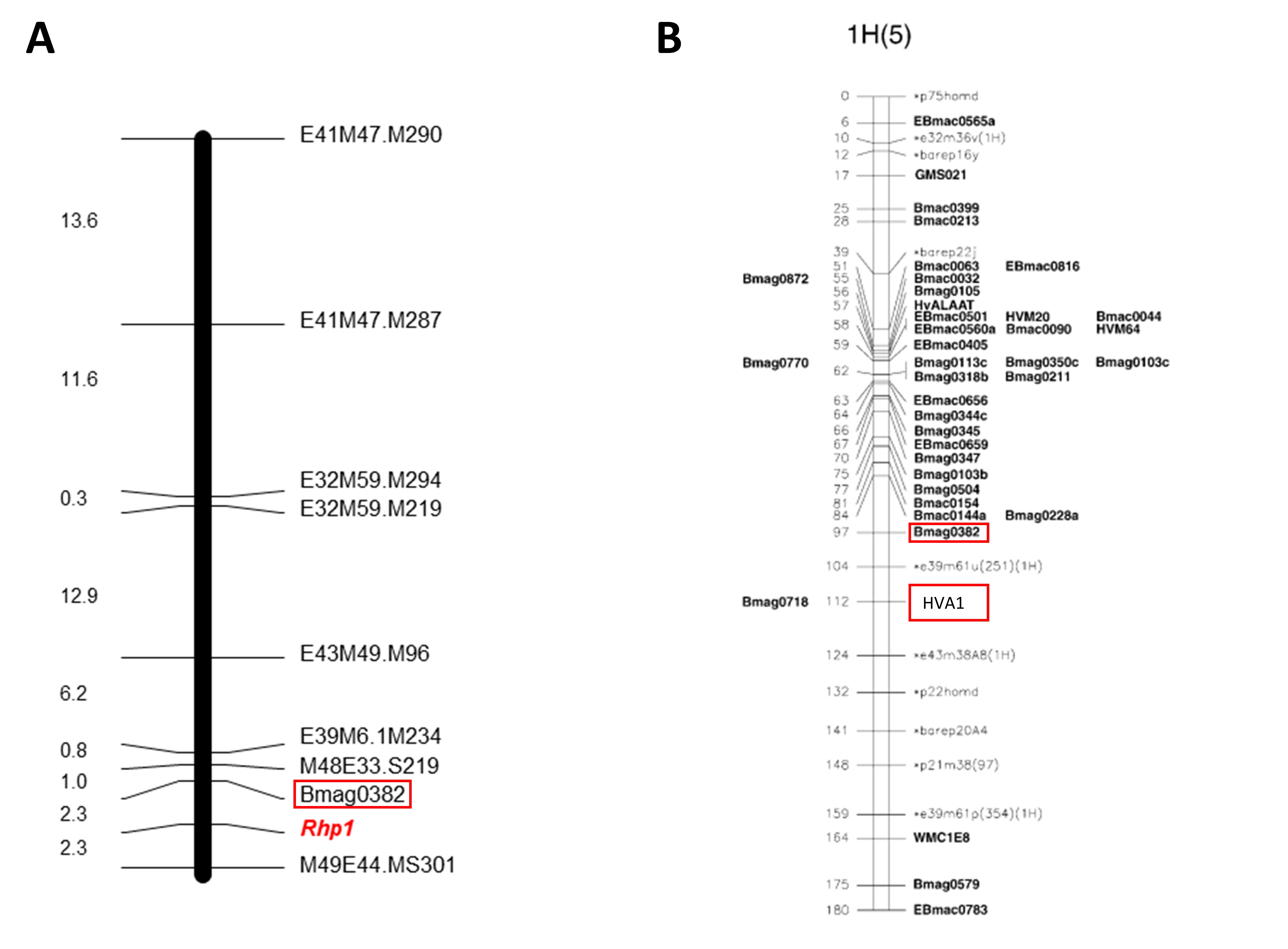

Supplement: Supplementary file 1 [file ijms-22-13411-s001.zip › SF_S3.jpg]

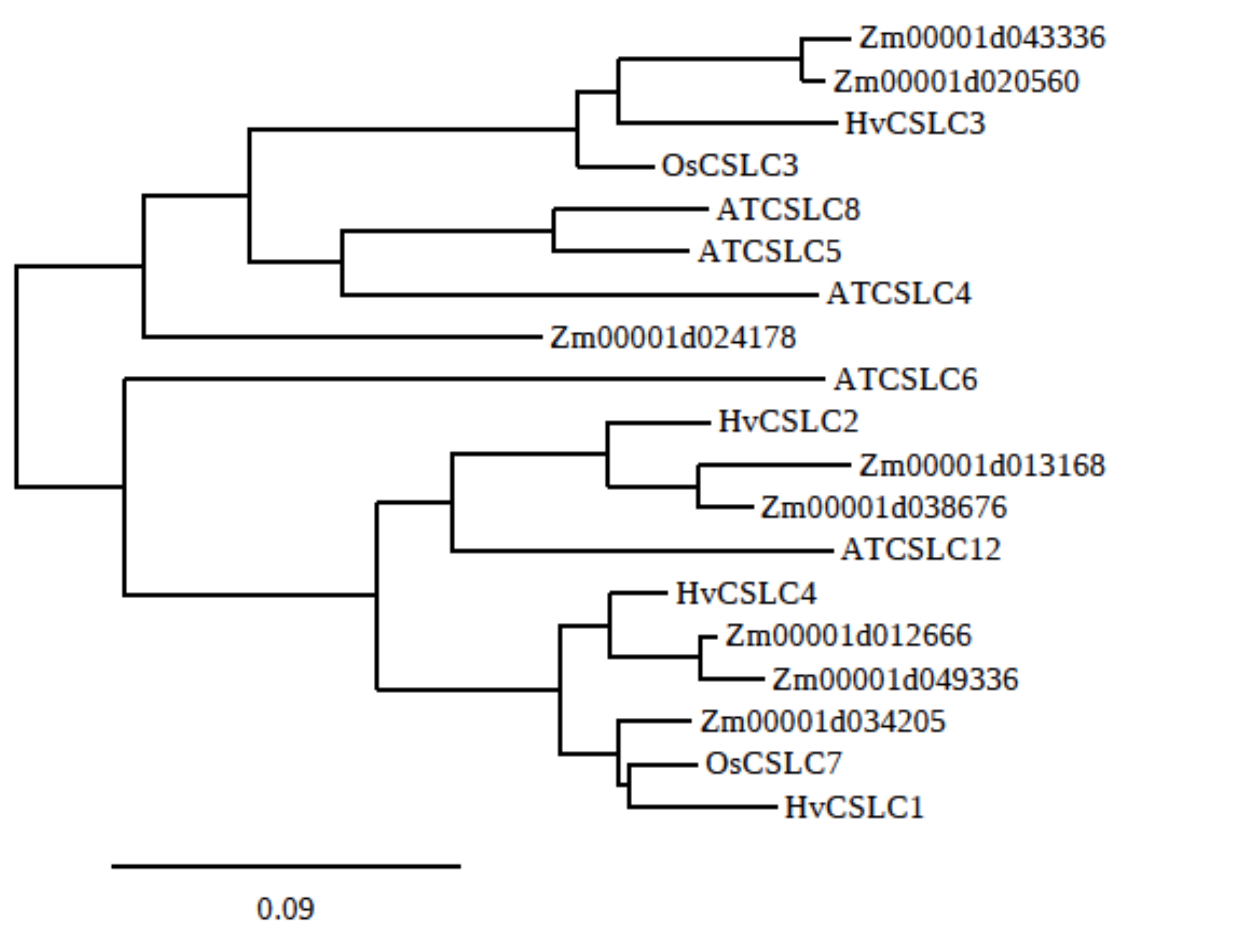

Supplement: Supplementary file 1 [file ijms-22-13411-s001.zip › SF_S4.jpg]

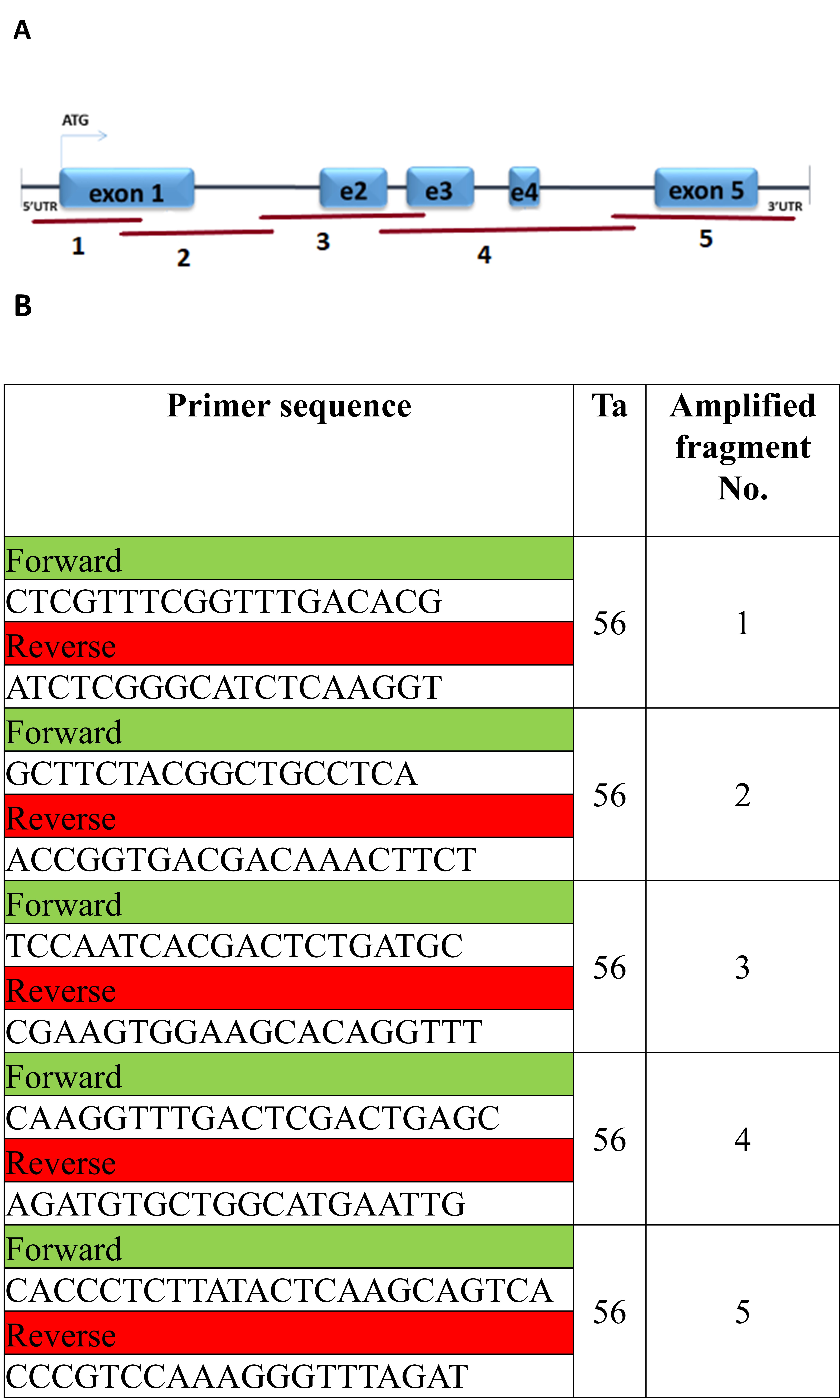

Supplement: Supplementary file 1 [file ijms-22-13411-s001.zip › SF_S5.jpg]

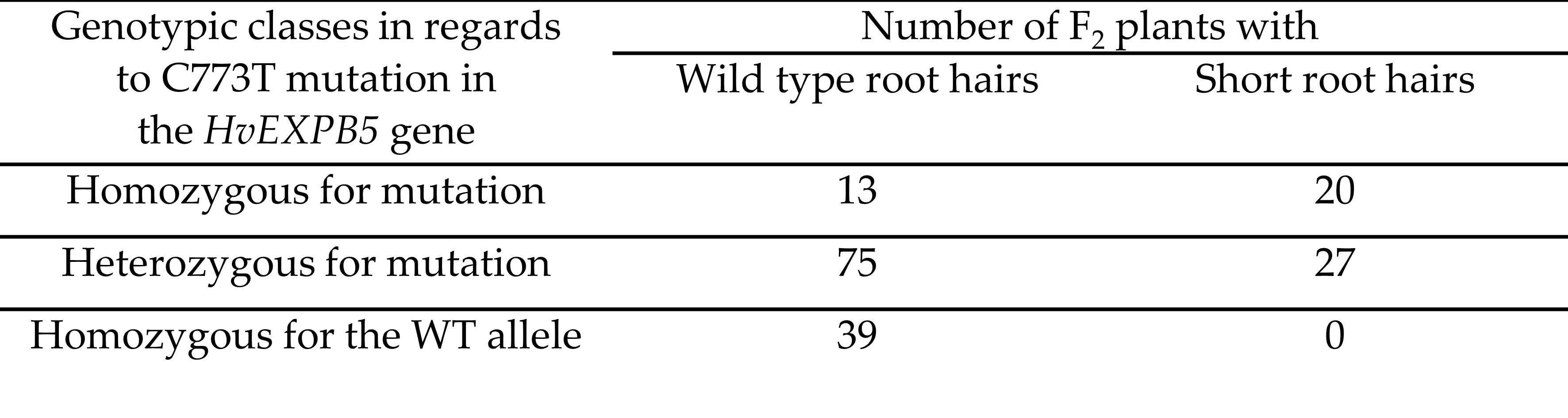

Supplement: Supplementary file 1 [file ijms-22-13411-s001.zip › Tab_S1.jpg]
